# Mutant p53^R273H^ disrupts PDPK1 homodimerization and contributes to PDPK1 activation

**DOI:** 10.1002/1878-0261.70341

**Published:** 2026-09-29

**Authors:** Mei Chee Lim, Alvin Kunyao Guo, Yoko Itahana, Srinivasaraghavan Kannan, Valerie Goh, Mingmin Ong, Terina Yan Wen Tay, Weiying Ye, Sabhashina Radha Krishnan, Jun Nishiyama, Chandra S Verma, Koji Itahana

**Affiliations:** ^1^ Programme in Cancer and Stem Cell Biology Duke‐NUS Medical School Singapore; ^2^ Bioinformatics Institute, Agency for Science Technology, and Research (A*STAR) Singapore; ^3^ Programme in Neuroscience and Behavioural Disorders Duke‐NUS Medical School Singapore; ^4^ School of Applied Science Temasek Polytechnic Singapore; ^5^ Department of Biological Sciences National University of Singapore Singapore; ^6^ School of Biological Sciences Nanyang Technological University Singapore

**Keywords:** AKT phosphorylation, homodimerization, interaction, mutant p53, PDPK1

## Abstract

The tumor suppressor p53 protein is frequently mutated in cancer, conferring oncogenic gain‐of‐function properties. One such mutant, p53^R273H^, is known to promote the activation of the PI3K/AKT pathway, but the underlying mechanism is unclear. Here, we demonstrated a direct interaction between p53^R273H^ and 3‐phosphoinositide‐dependent protein kinase 1 (PDPK1), an upstream kinase of AKT. Interestingly, this interaction was unique to p53^R273H^, as it was not seen with wild‐type p53 or other hotspot mutants such as p53^R175H^ and p53^R248W^. Mechanistically, our *in silico* analysis revealed that the R273H mutation exposed a hydrophobic region within the p53^R273H^ DNA‐binding domain (DBD), facilitating PDPK1 binding. This binding disrupted PDPK1 homodimerization, a known inhibitory mechanism of PDPK1 activity. In contrast, the PDPK1‐binding deficient mutant p53^F270A/R273H^ did not disrupt PDPK1 homodimerization. We propose that p53^R273H^ promotes AKT phosphorylation, potentially by enhancing PDPK1 homodimer dissociation. Supporting this, the small molecule PRIMA‐1^Met^ disrupted the p53^R273H^‐PDPK1 interaction, resulting in reduced AKT phosphorylation. These findings highlight a new gain‐of‐function role of p53^R273H^, suggesting that targeting the p53^R273H^‐PDPK1 interaction with PRIMA‐1^Met^ could dampen AKT phosphorylation in p53^R273H^‐expressing cancers.

## Graphical abstract

How mutant p53^R273H^ drives AKT signaling is unclear. We show that p53^R273H^, but not wild‐type, directly binds PDPK1 via a mutation‐dependent conformational change. This interaction disrupts inhibitory PDPK1 homodimerization and enhances AKT phosphorylation. PRIMA‐1^Met^‐mediated dissociation of the p53^R273H^‐PDPK1 complex restores PDPK1 homodimerization and attenuates AKT signaling, revealing a therapeutically targetable gain‐of‐function pathway in p53^R273H^‐expressing cancers.

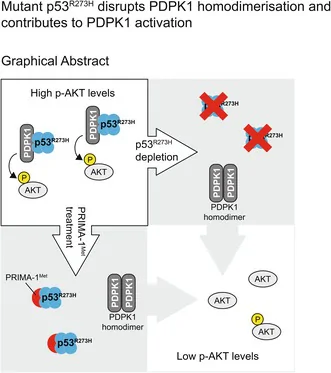

**Abbreviations**
2pFLIM: 2‐photon fluorescence lifetime imaging microscopy
aMD: accelerated molecular dynamics
DAPI: 4′,6‐diamidino‐2‐phenylindole
DAVID: database for annotation, visualization, and integrated discovery
DBD: DNA‐binding domain
FLIM: fluorescence lifetime imaging microscopy
FRET: Förster resonance energy transfer
GOF: gain‐of‐function
GST: glutathione S‐transferase
KEGG: Kyoto Encyclopedia of Genes and Genomes
MD: molecular dynamics
PDPK1: 3‐phosphoinositide‐dependent protein kinase 1
PI3K: phosphatidylinositol 3‐kinase
PLA: proximity ligation assay
PMSF: phenylmethylsulfonyl fluoride
PMT: photoelectron multiplier tubes
RNP: ribonucleoprotein
siRNA: small interfering RNA
STR: Short Tandem Repeat
TNFα: tumor necrosis factor α
VMD: visual molecular dynamics

## 1 Introduction

The tumor suppressor p53 protein is a transcription factor that is activated in response to cellular stresses such as DNA damage and oncogenic activation. Activated p53 induces the expression of genes involved in tumor suppression, including cell cycle arrest, senescence, DNA repair, and apoptosis [1, 2, 3, 4]. Not surprisingly, therefore, about 50% of all human cancers have mutations in the p53 protein. Approximately 75% of *TP53* mutations in human cancers are missense substitutions [5, 6]. Of these missense mutations, six hotspots occur within the DNA‐binding domain. These hotspot mutation sites were found to disrupt the DNA‐binding ability of p53. These mutant forms of p53 were expressed as full‐length proteins with single amino acid point mutations [7], unlike other tumor suppressor genes such as RB and APC, where mutations result in loss of expression of these tumor suppressor proteins. This led to the speculation that these p53 mutations have a selective advantage in cancer progression. Indeed, the studies of the knock‐in mice expressing hotspot *Trp53* mutations have provided robust evidence supporting the selective advantages of *Trp53* mutations in cancer progression [6, 8, 9]. In particular, *Trp53* ^*R270H/−*^ mice (mutation corresponds to R273H hotspot mutation in humans) were shown to develop a different spectrum of tumors compared to *Trp53* ^*−/−*^ mice. These tumors from *Trp53* ^*R270H/−*^ mice were also invasive and metastatic, further suggesting that the R273H mutation confers oncogenic gain‐of‐functions (GOFs) to the p53 protein rather than purely a loss of function [9]. Later studies showed that mutant p53^R273H^ dysregulates crucial signaling pathways and promotes oncogenic GOFs, including increased cell proliferation, survival, invasion, metastasis, and chemoresistance [5, 7, 9].

The phosphatidylinositol 3‐kinase (PI3K)/AKT pathway is aberrantly activated in human cancers [10]. Aberrant activation of the PI3K/AKT pathway drives several tumor‐associated processes, including cell proliferation, survival, invasion, metastasis, and chemoresistance [10, 11, 12] that overlap with the phenotypes of mutant p53. Several studies showed that mutant p53 promotes the activation of the PI3K/AKT pathway [13, 14, 15, 16]. The inhibition of the PI3K/AKT pathway can also dampen the mutant p53^R273H^ GOFs [17, 18]. These findings suggest that mutation of p53 and activation of the PI3K/AKT pathway are likely to co‐occur in cancers. Indeed, a recent study identified the PI3K/AKT/mTOR signaling pathway as the cooperating driver event in a mutant p53 mouse model that recapitulates metastatic breast cancer development [19]. Although p53 mutations and PI3K/AKT activation are frequently found in cancers, the mechanism by which mutant p53 activates the PI3K/AKT pathway is not fully understood. A clear understanding of the underlying mechanisms could potentially contribute to the development of effective therapeutic approaches targeting these cancers.

This study identified 3‐phosphoinositide‐dependent protein kinase 1 (PDPK1) as a novel interacting protein of mutant p53. We demonstrated that PDPK1 specifically interacts with p53^R273H^, but not with wild‐type p53 or other p53 mutants. Notably, we discovered that p53^R273H^ disrupts the PDPK1 homodimer configuration, a known inhibitory mechanism of PDPK1 activation. Finally, we showed that the p53^R273H^‐PDPK1 interaction can be disrupted using the small molecule PRIMA‐1^Met^, highlighting a potential therapeutic strategy for p53^R273H^‐expressing cancers.

## 2 Materials and methods

### 2.1 Cell lines and chemicals

U‐251MG (RRID:CVCL_0021) cell line was purchased from CLS Cell Lines Service GmbH (Cytion). SaOS‐2 (RRID:CVCL_0548), NCI‐H1299 (RRID:CVCL_0060), and HEK293T (RRID:CVCL_0063) cells were purchased from the American Type Culture Collection (ATCC). HCT116 TP53(−/−) (RRID:CVCL_HD97) knockout cell line was kindly provided by Dr. Bert Vogelstein at The Johns Hopkins University School of Medicine. All cell lines were cultured in Dulbecco's modified Eagle medium (DMEM, Thermo Fisher Scientific) supplemented with 10% fetal bovine serum (FBS, Thermo Fisher Scientific) and maintained in a 37 °C incubator with 5% CO_2_. Tumor necrosis factor α (TNFα) was purchased from Sigma‐Aldrich. PRIMA‐1^Met^ was purchased from MedChemExpress. Authentication was performed by 1^st^ Base using Short Tandem Repeat (STR) profiling analysis within 3 years of the time of use. Negative mycoplasma results were confirmed by PCR.

### 2.2 Constructs and cloning

pCMV5‐EGFP‐PDPK1, pCMV5‐HA‐PDPK1‐mCherry, and EGFP‐PDPK1‐∆PH constructs were kindly provided by Dr. Banafshe Larijani (University of Bath, UK). pCMV5‐HA‐PDPK1‐sREACh construct was generated by replacing the mCherry tag in pCMV5‐HA‐PDPK1‐mCherry with the sREACh tag. Full‐length *TP53* ^*R273H*^ and *PDPK1* were amplified from U‐251MG cells and cloned into the pcDNA3.1(+) vector (Thermo Fisher Scientific) with 3 × FLAG tagging to generate pcDNA3.1(+)‐p53^R273H^‐FLAG3 and pcDNA3.1(+)‐FLAG3‐PDPK1. PDPK1 was also subcloned into pET3E‐His vector to generate pET3E‐His‐PDPK1. CMV‐p53 wild‐type construct was kindly provided by Dr. Yanping Zhang (University of North Carolina at Chapel Hill, USA). The wild‐type p53 fragment was also cloned into pcDNA3.1(+) to generate pcDNA3.1(+)‐p53‐FLAG3. Point mutations F270A, R273H, R175H, and R248W were introduced using the PCR‐based HiFi site‐directed mutagenesis kit (New England Biolabs). Full‐length *TP53* ^*R273H*^ was also cloned into the pGEX2T vector (GE Healthcare). All cloned constructs were confirmed by DNA sequencing.

### 2.3 Adenovirus generation and infection

Adenoviruses expressing *TP53* ^*R273H*^ and *TP53* ^*F270A/R273H*^ were generated by cloning the respective coding regions of mutant *TP53* into AdEasy XL adenoviral vector (Agilent Technologies) as previously described [20]. Cells were seeded 24 h prior to the infection to reach 40% confluency. Cells were thoroughly washed twice with PBS before being cultured in serum‐free DMEM containing *TP53* ^*R273H*^ or *TP53* ^*F270A/R273H*^ adenovirus for 16 h. Next, an equal volume of virus‐free DMEM supplemented with 20% FBS was added to the cells for another 24 h. Subsequently, the cells were washed and lysed for immunoprecipitation.

### 2.4 Retrovirus generation and infection

To generate retroviruses carrying shRNAs against human *TP53*, *TP53* targeting sequence (GTAATCTACTGGGACGGAA) was cloned into a pSuper retro puro (Oligoengine). Retroviral transduction was performed as previously described [21]. Briefly, retrovirus vectors containing shRNA sequence together with amphotropic retroviral plasmids were cotransfected into HEK293T cells to generate viral particles. Cells at 50% confluence were infected with retrovirus overnight in the presence of 8 μg·mL^−1^ polybrene (Sigma‐Aldrich) and selected with 0.5 μg·mL^−1^ puromycin for 4 days after transduction.

### 2.5 Generation of U‐251MG p53 knockout cells

p53 knockout U‐251MG cells were generated using Cas9 Ribonucleoprotein (RNP) complex. *TP53* targeting crRNA (TCCACTCGGATAAGATGCTG, Integrated DNA Technologies, Inc.) was first annealed with tracrRNA‐ATTO550 (Integrated DNA Technologies, Inc.) before loading on the purified Cas9 protein (Integrated DNA Technologies, Inc.). The RNP complexes were electroporated into U‐251MG cells. Electroporated cells were sorted for positive ATTO550 fluorescence signals.

### 2.6 Western blotting analysis

Cells were washed three times with phosphate‐buffered saline (PBS) and lysed with 2% SDS lysis buffer (50 mM Tris/HCl pH 6.8, 10% glycerol, 2% SDS). Protein concentrations were quantified using DC Protein Assay Reagents (Bio‐Rad Laboratories). Equal amounts of total proteins were resolved in SDS‐polyacrylamide gel electrophoresis and transferred onto nitrocellulose membranes. Membranes were blocked with 5% nonfat milk in PBS containing 0.1% Tween‐20 and incubated with antibodies [mouse anti‐FLAG (Sigma‐Aldrich, #F1804), mouse anti‐p53 (Santa Cruz Biotechnology, #sc‐126), rabbit anti‐p53 (Santa Cruz Biotechnology, #sc‐6243), rabbit anti‐PDPK1 (Cell Signaling Technology, #3062), rabbit anti‐AKT (Cell Signaling Technology, #9272), rabbit anti‐phospho‐AKT (T308) (Cell Signaling Technology, #13038), mouse anti‐Actin (Merck Millipore, #MAB1501), mouse anti‐GST (Santa Cruz Biotechnology, #sc‐138), and rabbit anti‐MYC (Proteintech, #16286‐1‐AP)]. Detection of blots was done with ECL Prime Western Blotting Detection Reagent (Cytiva) for goat anti‐mouse or anti‐rabbit secondary antibodies conjugated with horseradish peroxidase (HRP) (Jackson ImmunoResearch) or done with Odyssey Infrared Imaging System (LI‐COR Biosciences) for DyLight 680‐ or 800‐fluorescent‐conjugated goat anti‐mouse or anti‐rabbit secondary antibodies (Thermo Fisher Scientific).

### 2.7 RNA interference

Small interfering RNA (siRNA) targeting *TP53* #1 (forward: GUAAUCUACUGGGACGGAAdTdT, reverse: UUCCGUCCCAGUAGAUUACdCdA), *TP53* #2 (forward: GGUGAACCUUAGUACCUAAdTdT, reverse: UUAGGUACUAAGGUUCACCdAdA), *PDPK1* #1 (forward: GCGGUUAGGCUGUGAGGAAdTdT, reverse: UUCCUCACAGCCUAACCGCdTdT), *PDPK1* #2 (forward: CGCCUAACAGGACGUAUUAdTdT, reverse: UAAUACGUCCUGUUAGGCGdTdG), or control siRNA (Qiagen, 1027310) were transfected into cells at a final concentration of 40 nM using the calcium phosphate transfection protocol as previously described [22].

### 2.8 FRET/FLIM analyses

Förster resonance energy transfer (FRET) imaging using a custom‐built 2‐photon fluorescence lifetime imaging microscopy (2pFLIM) was performed as described [23, 24]. In brief, EGFP was excited with a Ti:Sapphire laser (InSight X3, Coherent) tuned at a wavelength of 920 nm. The fluorescence emission was collected with an objective (60×, 1.0 numerical aperture, Olympus), divided with a dichroic mirror (565dcxr), and detected with photoelectron multiplier tubes (PMTs) placed after wavelength filters (ET520/60 M‐2P for green, ET620/60 M‐2p for red, Chroma). PMTs with low transfer time spread (H7422‐40p; Hamamatsu) were used for both red and green channels. Fluorescence lifetime images were obtained using a time‐correlated single‐photon counting board (TimeHarp 260 pico dual, Picoquant) controlled with custom software (FLIMage, Fluorescence Lifetime Imaging). Images were acquired at a resolution of 128 × 128 pixels at the frame rate of ~8 Hz and averaged over 24 frames. To obtain the EGFP fluorescence lifetime, all pixels in an image of a cell expressing EGFP‐PDPK1 were summed up. A homodimerization fraction was then calculated by fitting a fluorescence lifetime curve with a double‐exponential function convolved with the Gaussian pulse‐response function, which contains the mixture of non‐FRET and FRET fractions. Calculations are as described elsewhere [25, 26]. In brief, fluorescence lifetime is defined as the time constant of the fluorescence decay. The equation used is as follows:

$$F\left(t\right)=F_{0}\left[P_{D}\;\exp\left(-t/T_{D}\right)+P_{\mathrm{DA}}\;\exp\left(-t/T_{\mathrm{DA}}\right)\right]$$

where *F*_0_ is the constant, *T*_D_ is the fluorescence lifetime of the donor, EGFP‐PDPK1, and *T*_DA_ is the fluorescence lifetime of the positive control donor bound to the acceptor, EGFP‐sREACh. *P*_D_ and *P*_DA_ are the proportions of the donors that are free and bound to acceptor, respectively.

### 2.9 Molecular dynamics simulations of the 3D structures of p53^R273H^ DBD

Molecular Dynamics (MD) simulations were carried out on the p53 DNA‐binding domain (DBD) using the crystal structure of the DBD (residues 94 to 291, PDB ID: 2AHI) to generate the p53^WT^ DBD, p53^R273H^ DBD, p53^F270A/R273H^ DBD 3D structures. F270A is a control mutation that was speculated based on the conformational changes in the MD simulations of R273H to reverse the effect of the conformational change of R273H and was used to model the PDPK1‐p53^R273H^ DBD complexes later. The Xleap module of AMBER 18 [27] was used to prepare the systems for the MD simulations. Each simulation system was neutralized with appropriate numbers of counter ions, and each neutralized system was solvated in an octahedral box with TIP3P water molecules [28], leaving at least 10 Å between the solute atoms and the boundaries of the box. MD simulations were carried out with the pmemd.cuda module of the AMBER 18 package in combination with the ff14SB force field [29]. MD simulations were conducted in explicit solvent at 300 K. During the simulations, the long‐range electrostatic interactions were treated with the particle mesh Ewald method [30] using a real space cut off distance of 9 Å. The settle algorithm [31] was used to constrain bond vibrations involving hydrogen atoms, which allowed a time step of 2 fs during the simulations. Solvent molecules and counter ions were initially relaxed using energy minimization with restraints on the protein atoms. This was followed by unrestrained energy minimization to remove any steric clashes. Subsequently the system was gradually heated from 0 to 300 K using MD simulations with positional restraints (force constant: 50 kcal·mol^−1^·Å^−2^) on the protein atoms over a period of 0.25 ns allowing water molecules and ions to move freely followed by gradual removal of the positional restraints and a 2 ns unrestrained equilibration at 300 K. The resulting system was used as the starting structure for the production phase, and three independent (using different initial random velocities) MD simulations were carried out for 100 ns each. To further explore conformational sampling, the final snapshots from each of the triplicate 100 ns simulations of the DBDs of p53^WT^, p53^R273H^, and p53^F270A/R273H^ were each used to run triplicate 5 μs of accelerated molecular dynamics simulations (aMD) [32]. aMD simulations were performed using the ‘dual boost’ version. The average dihedral and total potential energies computed during 100 ns classical molecular dynamics simulations were used to derive the aMD parameters (EthreshP, alphaP, EthreshD, alphaD). Simulation trajectories were visualized using Visual Molecular Dynamics (VMD) [33], and figures were generated using PyMOL [34].

Models of the putative structural complexes between the p53 DBD and PDPK1 were generated using the experimental atomic structure (PDPK1 kinase domain) and snapshots from molecular dynamics simulations of p53^R273H^ DBD carried out in‐house as abovementioned. The crystal structure of the kinase domain of PDPK1 (residues 76 to 359, PDB ID: 4RRV), and the models of the p53^R273H^ DBD and p53^F270A/R273H^ DBD generated from MD simulation were used for *in silico* docking calculations. To generate the models of the complexes between PDPK1 and p53DBD, we used the program HADDOCK, adopting the standard parameters [35]. To generate the PDPK1‐p53^R273H^ DBD complex, the binding site on PDPK1 was defined as the PIF pocket (residues: 114–135, 145–153), and in p53^R273H^ DBD, the region (residues 109–114) that was exposed during the MD simulations was defined as the binding site. The docking poses that were obtained were analyzed visually, and the docked pose with the best score was further refined using MD simulations.

### 2.10 Mass spectrometry, KEGG pathway enrichment, and CRAPome

Mutant p53^R273H^‐interacting proteins were identified using mass spectrometry as described [21]. The data were analyzed using GPM software (https://gpmdb.thegpm.org/) with default parameter settings. Proteins with the expectation values log(e) of −1.0 [36] or lower were used for downstream analysis. A hundred and fifty‐one proteins were identified after background subtraction. The identified proteins were subjected to Kyoto Encyclopedia of Genes and Genomes (KEGG) pathway enrichment analysis using the Database for Annotation, Visualization, and Integrated Discovery (DAVID). Twelve proteins in the top enriched PI3K‐AKT pathway were queried on the CRAPome database [37] (https://reprint‐apms.org/) to evaluate specific interactions.

### 2.11 Immunoprecipitation

Cells were washed three times with PBS and lysed with 0.1% NP‐40 lysis buffer (50 mm Tris/HCl pH 7.5, 150 mm NaCl, 0.1% NP‐40, 50 mm NaF, 1 mm Na_3_VO_4_, 1 mm phenylmethylsulfonyl fluoride (PMSF), EDTA‐free protease inhibitor cocktail). Cell lysates were precleared using Sepharose CL‐4B beads (Sigma‐Aldrich) for 30 min at 4 °C, and 1 mg of total proteins were immunoprecipitated with antibodies [mouse anti‐FLAG (Sigma‐Aldrich, #F1804), goat IgG (Santa Cruz Biotechnology, #sc‐34665), goat anti‐p53 (Santa Cruz Biotechnology, #sc‐6243‐G), FLAG antibody‐conjugated beads (Sigma‐Aldrich, #F2426) or mouse anti‐MYC (Santa Cruz Biotechnology, #sc‐40)] for 16 to 24 h at 4 °C with constant rotation. For lysates incubated with antibodies, 15 μL of 50% Protein A/G agarose bead slurry (Thermo Fisher Scientific) was subsequently added and incubated for 2 h. Beads were then washed three times with 0.1% NP‐40 lysis buffer before being subjected to western blot analysis.

### 2.12 Indirect immunofluorescence

Cells were washed once with PBS and fixed for 10 min with 4% paraformaldehyde (Sigma‐Aldrich) in PBS without agitation. Cells were rinsed twice with PBS and permeabilized with 0.2% Triton X‐100 in PBS for 5 min at 4 °C. Next, cells were washed twice with PBS and then blocked with 0.5% BSA in PBS for 60 min at room temperature with gentle rocking. The blocking solution was aspirated and replaced with the blocking solution containing mouse anti‐p53 (Santa Cruz Biotechnology, #sc‐126), rabbit anti‐PDPK1 (Atlas Antibodies, #HPA068961 or #HPA035199), and incubated at 4 °C overnight with gentle rocking. Cells were then washed three times with PBS, 10 min for each wash with gentle rocking at room temperature. Cells were incubated with Alexa Fluor 488‐conjugated donkey anti‐mouse or anti‐rabbit IgG (Jackson ImmunoResearch) for 1 h in the dark with gentle rocking at room temperature. Cells were rinsed once with PBS and replaced with PBS containing 1 μg·mL^−1^ of DAPI (4′,6‐diamidino‐2‐phenylindole) (Nacalai Tesque) and incubated for 2 min at room temperature with gentle rocking. Cells were washed 3 times with PBS and covered with a glass coverslip with 1 drop of DAKO mounting solution (Agilent Technologies). Immunofluorescence was detected using the Olympus IX71 inverted microscope, and resulting images were analyzed using the ImageJ software (version 2.0.0‐rc‐69/1.52n; National Institutes of Health).

### 2.13 Duolink proximity ligation assay (PLA)

Protein–protein interaction was demonstrated using the Duolink II detection kit according to the manufacturer's guidelines (Sigma‐Aldrich). Briefly, cells were fixed with 4% paraformaldehyde, permeabilized, blocked, and incubated with mouse anti‐p53 (Santa Cruz Biotechnology, #sc‐126) and rabbit anti‐FLAG (Proteintech, #80010‐1‐RR; probing for FLAG‐tagged PDPK1) primary antibodies. Anti‐mouse PLUS and anti‐rabbit MINUS probes (Sigma‐Aldrich) were used as secondary antibodies. Ligation and amplification reactions were performed according to the manufacturer's instructions. After PLA staining, cells were incubated with Alexa Fluor 594‐conjugated donkey anti‐rabbit IgG (Jackson ImmunoResearch) and CF680R‐conjugated donkey anti‐mouse IgG (Biotium) for 1 h in the dark with gentle rocking at room temperature. Cells were rinsed once with PBS and replaced with PBS containing 1 μg·mL^−1^ of DAPI (Nacalai Tesque) and incubated for 2 min at room temperature with gentle rocking. Cells were washed 3 times with PBS and covered with a glass coverslip with 1 drop of DAKO mounting solution (Agilent Technologies). Images were acquired using a confocal microscope (Carl Zeiss LSM 710) equipped with an oil‐immersion objective lens (NA 1.40, ×63; Plan Apochromat, Carl Zeiss) and zen 2010 software (version 6.0.0.486; Carl Zeiss). The resulting images were analyzed using the imagej software (version 2.0.0‐rc‐69/1.52n; National Institutes of Health).

### 2.14 Recombinant protein expression and purification

Expression of GST‐p53^R273H^ and 6xHis‐PDPK1 was induced in *Escherichia coli* BL21(DE3) cells with 1 mm IPTG (isopropyl‐β‐d‐thiogalactopyranoside) for 4 h at 37 °C. Recombinant proteins were purified as previously described [20]. Briefly, bacterial cells expressing GST fusion proteins were lysed in sonication buffer (50 mm Tris/HCl [pH 8.0], 0.5 M NaCl, 10% glycerol, 1% Triton X‐100, 1 mm PMSF, 1 mm DTT, protease inhibitors) and incubated for 10 min at room temperature. Lysozyme was then added to a final concentration of 1 mg·mL^−1^, and the mixture was rotated for 30 min at room temperature. Subsequently, sonication was performed on ice using six cycles of 10 s on and 10 s off. The lysate was centrifuged at 12,000 × ***g*** for 10 min at 4 °C. GST fusion proteins were then enriched using Pierce™ Glutathione Agarose beads (Thermo Fisher Scientific) for 2 h at 4 °C. The beads were washed three times with washing buffer (50 mm Tris/HCl [pH 8.0], 1 mm DTT, 5 mm MgCl_2_, 100 mm NaCl, 1% Triton X‐100, and 1 mm PMSF) and used in the bead‐conjugated form for pulldown assay. Bacterial cells expressing 6xHis‐PDPK1 were processed similarly to the GST fusion proteins and enriched using HisPur™ Ni‐NTA Resin (Thermo Fisher Scientific), eluted with 500 mM imidazole, and dialyzed against PBS.

### 2.15 *In vitro* glutathione S‐transferase (GST) pulldown assay

GST‐tagged p53^R273H^ or GST proteins immobilized on Pierce™ Glutathione Agarose beads (Thermo Fisher Scientific) were incubated with 6 × His‐PDPK1 protein for 2 h at 4 °C with constant rotation. Beads were then washed five times with 0.1% NP‐40 lysis buffer (50 mm Tris/HCl pH 7.5, 0.1% NP‐40, 150 mm NaCl, 50 mm NaF, 1 mm Na_3_VO_4_, 1 mm PMSF, EDTA‐free protease inhibitor cocktail). The bound proteins were eluted in SDS/PAGE loading buffer for western blot analysis.

### 2.16 *In vitro* PDPK1 kinase assay

PDPK1 kinase assay was performed using Chemi‐Verse™ kit (BPS Bioscience) according to the manufacturer's instructions, with minor modifications. Recombinant GST‐PDPK1 was pre‐incubated with either recombinant GST or GST‐p53^R273H^ on ice for 1 h. Following incubation, the kinase reaction was initiated by adding the substrate (PDKtide) and ATP. The activity of PDPK1 kinase was quantified by measuring ADP production using the ADP‐Glo™ kinase assay kit (Promega).

### 2.17 TNFα and PRIMA‐1^Met^ treatments

U‐251MG cells were washed three times with PBS and then treated for 24 h with either DMSO (vehicle control), 10 ng·mL^−1^ of TNFα as described [38], 0.125 mm PRIMA‐1^Met^, or a combination of 10 ng·mL^−1^ of TNFα and 0.125 mm PRIMA‐1^Met^. All treatments were performed in DMEM containing 0.1% FBS.

### 2.18 Statistical analyses

A two‐tailed unpaired Student's *t*‐test was used to determine significant differences for all the data unless otherwise stated. One‐way ANOVA with *post hoc* Tukey's test was used to determine significant differences for FRET/FLIM analysis (Figs 3C and 5C). All statistical analyses were conducted using GraphPad Prism 9.3.1 software.

## 3 Results

### 3.1 Mutant p53^R273H^ binds to PDPK1

Given that mutant p53 loses its DNA‐binding ability, its oncogenic properties may be attributed to interactions with other proteins [39]. Our previous screening identified 151 potential interacting proteins of mutant p53 [21] (Fig. 1A). The presence of known mutant p53 interacting proteins, such as MDM2, NRD1, and HSP90, within this identified list validated our screening method. KEGG pathway analysis of the identified mutant p53 interacting proteins revealed that the top enriched pathway was the PI3K‐AKT signaling pathway (Fig. 1B), which consists of twelve p53^R273H^ interacting proteins (Table S1). Since PDPK1 is a direct upstream kinase of AKT with the lowest CRAPome value (Table S1), we decided to focus our study on the p53^R273H^‐PDPK1 interaction among the twelve interacting proteins in the AKT signaling pathway.

**Fig. 1:**
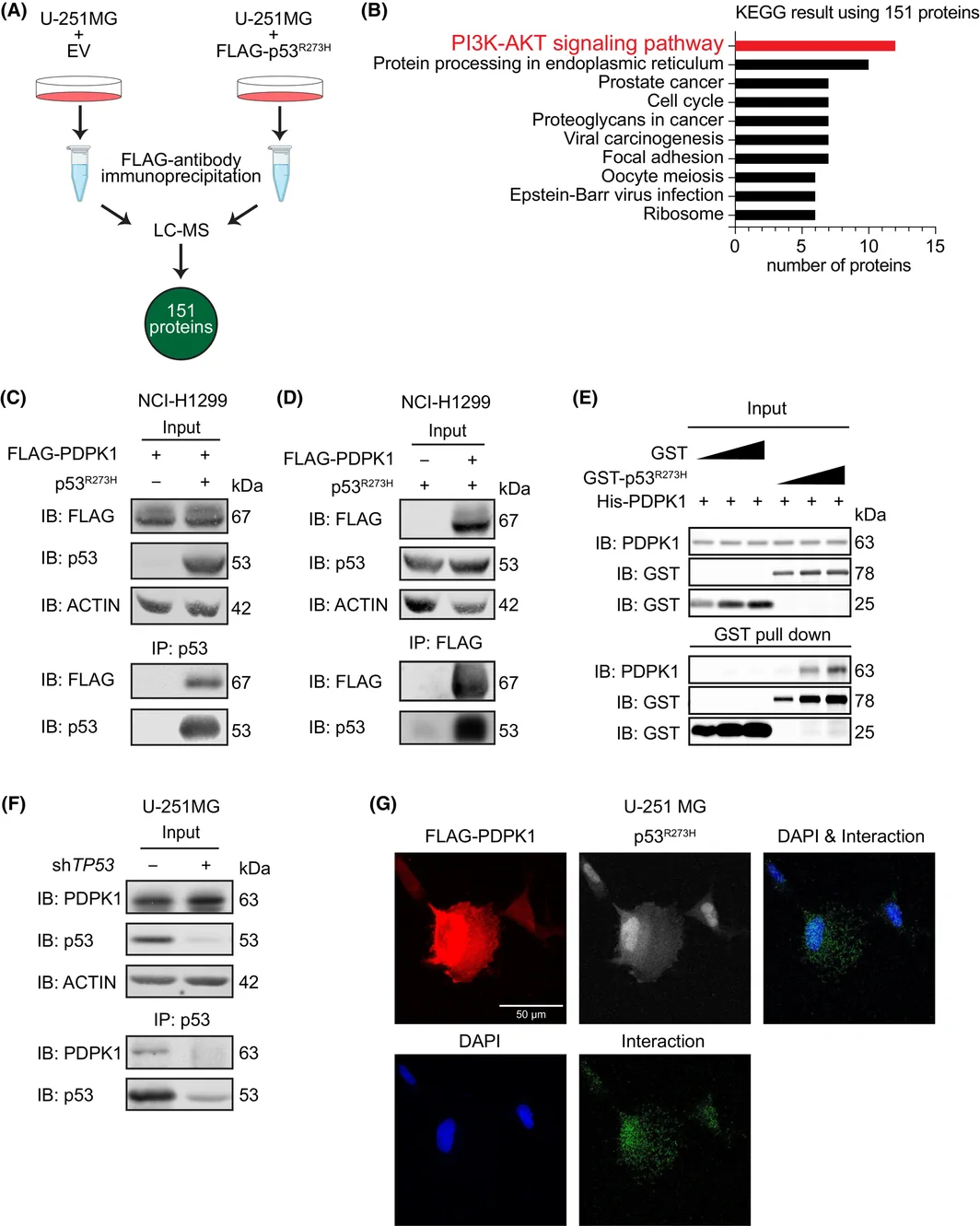
Mutant p53^R273H^ binds to PDPK1. (A) A schematic workflow of the screening approach. Lysates from U‐251MG cells expressing p53^R273H^‐FLAG were immunoprecipitated with anti‐FLAG antibody, and the immunoprecipitated proteins were analyzed by mass spectrometry. (B) KEGG pathway analysis of the 151 p53^R273H^‐interacting proteins using DAVID (Database for Annotation, Visualization and Integrated Discovery). The top 10 enriched pathways are shown. (C and D) Co‐immunoprecipitation of exogenous p53^R273H^ and FLAG‐PDPK1. Lysates of NCI‐H1299 cells transfected with the indicated plasmids were immunoprecipitated with anti‐p53 (C) or anti‐FLAG (D) antibody and analyzed by western blot using the indicated antibodies (both panels are representative of *n* = 3 independent experiments). (E) GST pulldown assay. Purified 6 × His‐PDPK1 was incubated with GST or GST‐p53^R273H^ proteins immobilized on glutathione agarose beads and analyzed by western blot using the indicated antibodies (representative of *n* = 4 independent experiments). (F) Co‐immunoprecipitation of endogenous p53^R273H^ and PDPK1. Lysates of U‐251MG cells transduced with shControl‐ or sh*TP53*‐expressing retroviruses were immunoprecipitated with anti‐p53 antibody and analyzed by western blot using the indicated antibodies (representative of *n* = 5 independent experiments). (G) Proximity ligation assay (PLA) in U‐251MG cells. Cells transfected with FLAG‐PDPK1 were fixed, permeabilized, and stained using anti‐p53 and anti‐FLAG antibodies for PLA. Confocal microscopy images show FLAG‐PDPK1 (red), p53^R273H^ (gray), nuclei (DAPI, blue), and green fluorescence signals indicating positive PLA interactions (representative of *n* = 3 independent experiments). Scale bar = 50 μm (applies to all the images in the panel).

To confirm the p53^R273H^‐PDPK1 interaction, co‐immunoprecipitation assays were performed using the *p53*‐null NCI‐H1299 cell line, with ectopically expressed PDPK1 and p53^R273H^. The pulldown of p53^R273H^ co‐immunoprecipitated PDPK1 (Fig. 1C). Ectopically expressed p53^R273H^‐FLAG also co‐immunoprecipitated endogenous PDPK1 in the *TP53*‐null SaOS‐2 cell line (Fig. S1A). Consistently, the reciprocal pulldown of FLAG‐PDPK1 co‐immunoprecipitated ectopically expressed p53^R273H^ (Fig. 1D). Next, we tested if PDPK1 interacts directly with p53^R273H^. GST pulldown of purified GST‐p53^R273H^ precipitated purified His‐PDPK1, demonstrating a direct interaction (Fig. 1E).

To examine whether endogenous p53^R273H^ and PDPK1 interact, we performed a co‐immunoprecipitation assay using U‐251MG cells, which carry the *TP53* ^*R273H/R273H*^ mutation. The pulldown of endogenous p53^R273H^ co‐immunoprecipitated endogenous PDPK1 in control cells, but not in p53^R273H^‐depleted cells (Fig. 1F). Their binding specificity was further confirmed by using the nonspecific IgG isotype control (Fig. S1B).

Subsequently, we investigated the cellular localization of their interaction using the proximity ligation assay (PLA). Although the anti‐PDPK1 antibody worked well for western blot analysis, it did not specifically detect endogenous PDPK1 in U‐251MG cells (Fig. S2). Therefore, U‐251MG cells ectopically expressing FLAG‐PDPK1 were used for PLA. Immunofluorescence staining revealed close proximity between FLAG‐PDPK1 and endogenous p53^R273H^ in both the nucleus and cytoplasm (Fig. 1G). Taken together, these data strongly indicate that p53^R273H^ directly binds to PDPK1.

### 3.2 The R273H mutation in p53 potentially creates a motif that enables p53^R273H^ to bind to PDPK1

p53^R273H^ is classified as a contact mutant, exhibiting localized structural alterations within the DNA‐binding domain (DBD) while maintaining the overall conformation of the p53 protein [40]. This structural similarity to wild‐type p53 prompted us to investigate whether PDPK1 also binds to the wild‐type form. Interestingly, however, unlike p53^R273H^, wild‐type p53 did not co‐immunoprecipitate with PDPK1 (Fig. 2A and Fig. S1C). These findings suggest that the single amino acid substitution, arginine to histidine at position 273, may induce local structural changes that create a novel binding motif for PDPK1.

**Fig. 2:**
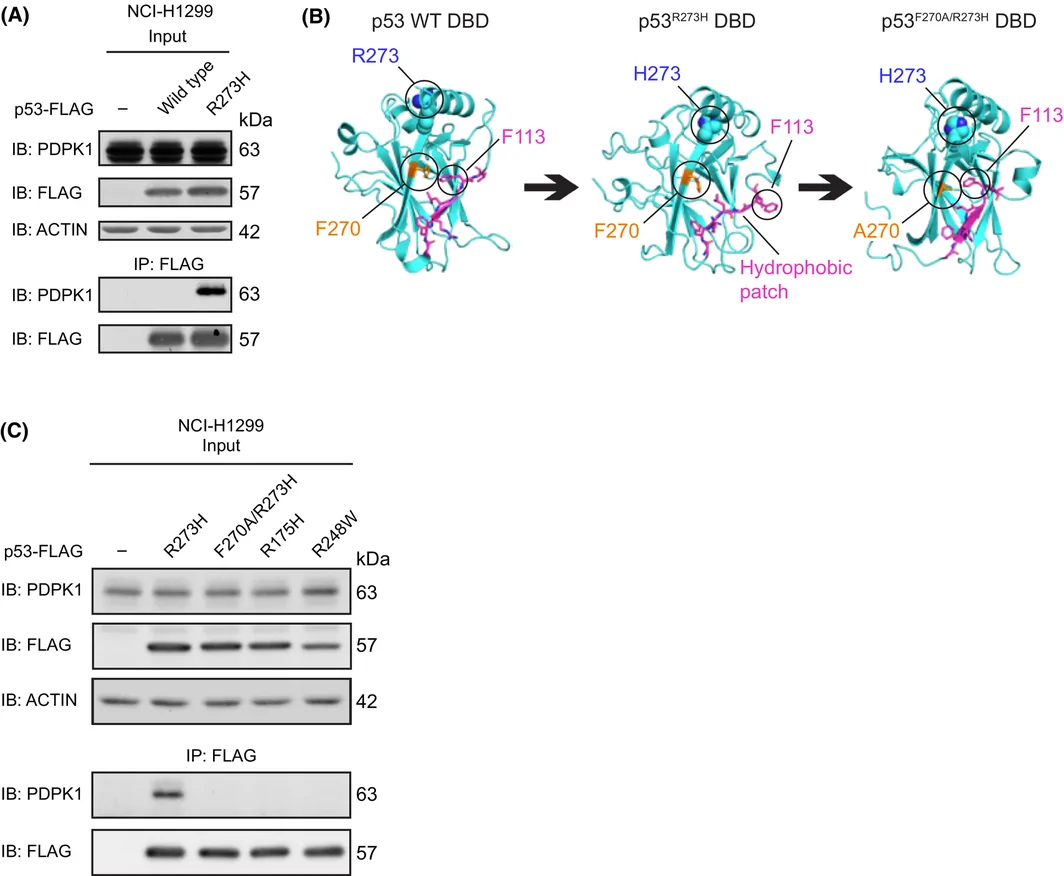
F270A mutation in p53^R273H^ disrupts PDPK1 binding. (A) Lysates of NCI‐H1299 cells transfected with the indicated plasmids were immunoprecipitated with anti‐FLAG antibody and analyzed by western blot using the indicated antibodies (representative of *n* = 2 independent experiments). Additional validation is provided in Fig. S1C. (B) Molecular Dynamics (MD) simulations on the p53 DNA binding domain (DBD) structural changes following point mutations at specific amino acid positions. Amino acid positions 270 (in orange), 273 (in blue), and 109–114 (in magenta) are shown. (C) Lysates of NCI‐H1299 cells transfected with the indicated plasmids were immunoprecipitated with anti‐FLAG antibody and analyzed by western blot using the indicated antibodies (representative of *n* = 3 independent experiments).

To explore this possibility, we conducted Molecular Dynamics (MD) simulations of the apo DBD of both wild‐type p53 and p53^R273H^. The MD simulation revealed that the R273H mutation caused a hydrophobic‐rich patch (109FRLGFL114, highlighted in magenta) to shift from the protein core to the surface of the p53^R273H^ DBD (Fig. 2B), whereas this region remains buried in the wild‐type DBD. This suggests that the mutation exposes a potential PDPK1‐binding site.

To test the importance of this hydrophobic patch for the interaction between p53^R273H^ and PDPK1, we first examined whether an additional mutation in p53^R273H^ DBD could revert this hydrophobic patch to its buried position, as observed in the wild‐type DBD. Visualization of the MD trajectories of wild‐type p53 DBD showed that F113 stacks against F270, keeping the hydrophobic patch buried (Fig. 2B), in contrast to the exposed F113 and L114 residues in p53^R273H^ DBD (Fig. 2B). Interestingly, MD simulations showed that the introduction of the F270A point mutation in p53^R273H^ DBD could revert this hydrophobic patch to its buried position, as observed in the wild‐type DBD (Fig. 2B). We therefore introduced the F270A mutation into p53^R273H^ to bury the hydrophobic patch without changing any of its residues 109FRLGFL114.

To experimentally validate this prediction, we first confirmed that the double mutant p53^F270A/R273H^ localized in the nucleus in a manner similar to the p53^R273H^ (Fig. S3). Next, we performed immunoprecipitation assays to compare the binding of p53^R273H^ and p53^F270A/R273H^ with PDPK1. The results demonstrated that p53^F270A/R273H^ failed to bind PDPK1 in both NCI‐H1299 and SaOS‐2 cells (Fig. 2C and Fig. S1D), suggesting that the absence of binding between PDPK1 and p53^F270A/R273H^ was likely due to the reburial of the hydrophobic patch. Additionally, other p53 mutants such as p53^R175H^ and p53^R248W^ did not bind to PDPK1 (Fig. 2C). Consistent with these findings, p53^R175H^ and p53^R248W^ failed to exhibit clear PLA signals with PDPK1, in contrast to the strong signals observed with p53^R273H^ in U‐251MG p53 knockout cells (Figs S4 and S5).

Taken together, these results suggest that the R273H mutation in p53 creates a novel motif that allows binding to PDPK1, potentially contributing to a gain‐of‐function specific to p53^R273H^.

### 3.3 Mutant p53^R273H^ disrupts PDPK1 homodimerization

Studies have demonstrated that dissociation of PDPK1 homodimer is crucial for its activation [41, 42]. To investigate whether p53^R273H^ binding to PDPK1 affects its activity, we examined the effect of p53^R273H^ on PDPK1 homodimerization. PDPK1 homodimerization was assessed using Förster resonance energy transfer (FRET) coupled with 2‐photon fluorescence lifetime imaging microscopy (2pFLIM), a method previously used to study protein dimerization, including PDPK1 [42]. We generated the constructs to express EGFP‐tagged PDPK1 and sREACh‐tagged PDPK1. PDPK1 homodimerization was quantified by measuring the reduction in EGFP fluorescence lifetime in the presence of the acceptor sREACh, which occurs as a result of FRET [43] (Fig. 3A). When PDPK1 homodimerizes (panel top left in Fig. 3A), EGFP will be close to sREACh where EGFP (donor) transfers its energy to sREACh (acceptor), and FRET occurs. In the FRET state, the EGFP has a shorter fluorescence decay time (short EGFP lifetime). In NCI‐H1299 cells expressing both EGFP‐PDPK1 and PDPK1‐sREACh, we observed a decreased EGFP fluorescence lifetime compared to cells expressing EGFP‐PDPK1 alone (Fig. 3B, panels a and b), suggesting an increased homodimerization fraction between EGFP‐PDPK1 and PDPK1‐sREACh (Fig. 3C, columns 1 and 2). To confirm that this observation reflected homodimerization, we expressed EGFP‐PDPK1 with a PH domain deletion (∆PH), which is essential for PDPK1 homodimerization [42]. Expression of EGFP‐PDPK1‐∆PH reduced the homodimerization fraction (Fig. 3B, panels b and c, and Fig. 3C, columns 2 and 3). Next, we introduced p53^R273H^ or the PDPK1‐binding deficient mutant p53^F270A/R273H^ to examine their effect on the EGFP‐PDPK1/PDPK1‐sREACh homodimerization fraction. We observed a decreased homodimerization fraction in p53^R273H^‐expressing cells (Fig. 3B, panels b and d, and Fig. 3C, columns 2 and 4), but not in p53^F270A/R273H^‐expressing cells (Fig. 3B, panels b and e, and Fig. 3C, columns 2 and 5). Taken together, these data suggest that p53^R273H^ interacts with PDPK1 to disrupt its homodimerization, potentially affecting PDPK1's activity.

**Fig. 3:**
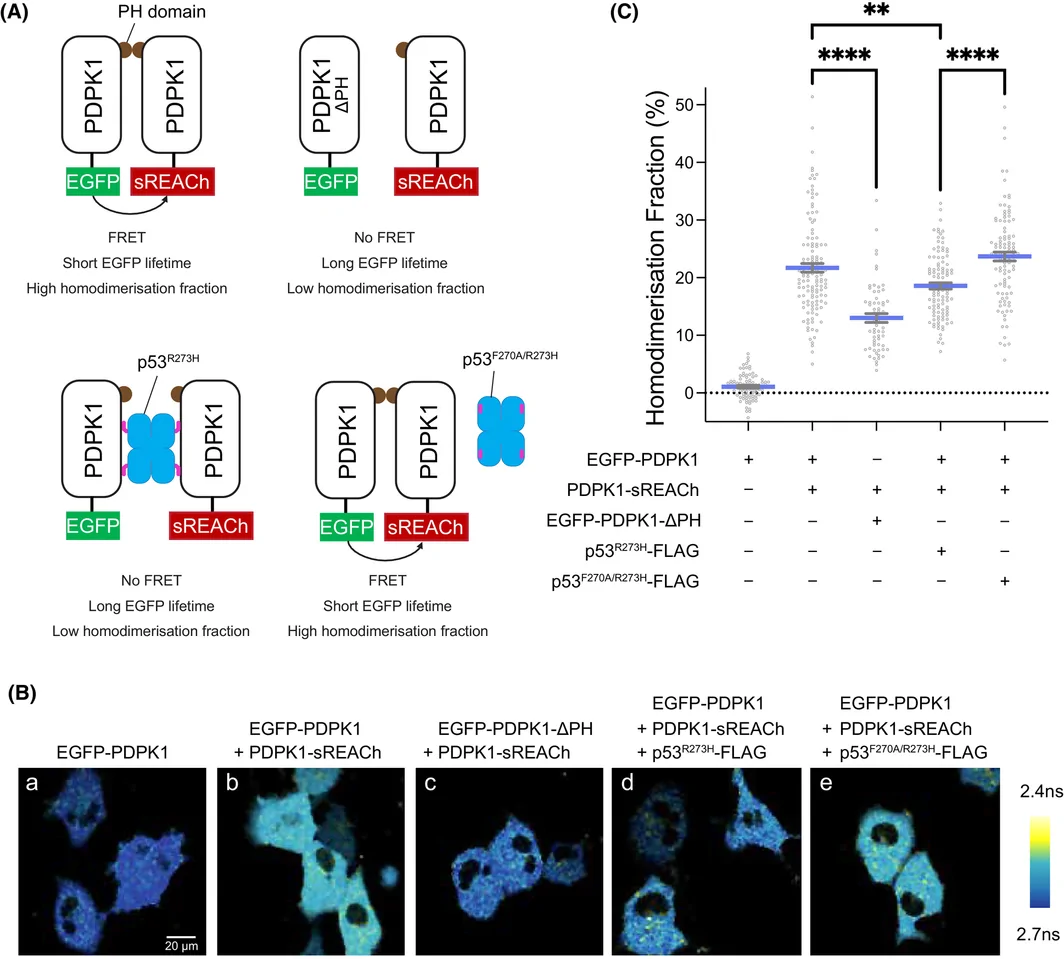
Mutant p53^R273H^ disrupts PDPK1 homodimerization. (A) A schematic illustration of FRET from EGFP‐PDPK1 to PDPK1‐sREACh under different conditions that affect PDPK1 homodimerization and alter the EGFP donor fluorescence lifetime. (B) Fluorescence lifetime images of NCI‐H1299 cells transfected with the indicated plasmids. Pseudo‐color represents the EGFP fluorescence lifetime; blue indicates longer lifetimes, and warmer colors (yellow) indicate shorter lifetimes due to FRET, reflecting increased PDPK1 homodimerization (representative of *n* = 3 independent experiments). Scale bar = 20 μm (applies to all the images in the panel). (C) Quantification of FRET‐FLIM analysis. Fluorescence lifetime images were acquired using a time‐correlated single‐photon counting board controlled by custom software. The fluorescence signals from all pixels within each cell expressing EGFP‐PDPK1 were summed to generate an EGFP fluorescence lifetime curve. The homodimerization fraction, defined as the proportion of EGFP‐PDPK1 molecules undergoing FRET with PDPK1‐sREACh, was then calculated by fitting the fluorescence lifetime curve with a double‐exponential function convolved with a Gaussian pulse‐response function, incorporating a mixture of non‐FRET and FRET fractions. A higher homodimerization fraction indicates increased PDPK1‐PDPK1 dimer formation. Gray circles represent individual cells (number of cells = 87, 126, 62, 105, 106 from left to right). Blue horizontal lines and gray error bars represent the mean homodimerization fraction ± SEM for each group (representative of *n* = 3 independent experiments). Statistical analysis was performed using a one‐way ANOVA with *post hoc* Tukey's test. **P value < 0.01, ****P value < 0.0001.

### 3.4 Mutant p53^R273H^ enhances PDPK1 activation

PDPK1 is an upstream kinase of AKT and directly phosphorylates threonine residue 308 (T308) [44, 45]. Indeed, PDPK1 knockdown in U‐251MG cells reduced AKT phosphorylation under our cell culture conditions (Fig. 4, lanes 4–5). We also observed that p53^R273H^ knockdown reduced AKT phosphorylation (Fig. 4, lanes 2–3). These results suggest that p53^R273H^ promotes PDPK1 activation, leading to increased AKT phosphorylation. To determine whether p53^R273H^ directly modulates PDPK1 kinase activity, we performed *in vitro* kinase activity assays using recombinant GST‐p53^R273H^ and GST‐PDPK1 (Fig. S6). A marginal increase in PDPK1 activity was observed upon the addition of p53^R273H^. As an *in vitro* system lacks the spatial organization or biochemical complexity required to fully recapitulate the cellular context, it remains difficult to precisely determine the extent to which p53^R273H^ directly contributes to PDPK1 kinase activity. Nevertheless, our combined data (Figs 3, 4 and Fig. S6) suggest that the disruption of the p53^R273H^‐PDPK1 complex could potentially diminish PDPK1 activity and, consequently, decrease AKT phosphorylation.

**Fig. 4:**
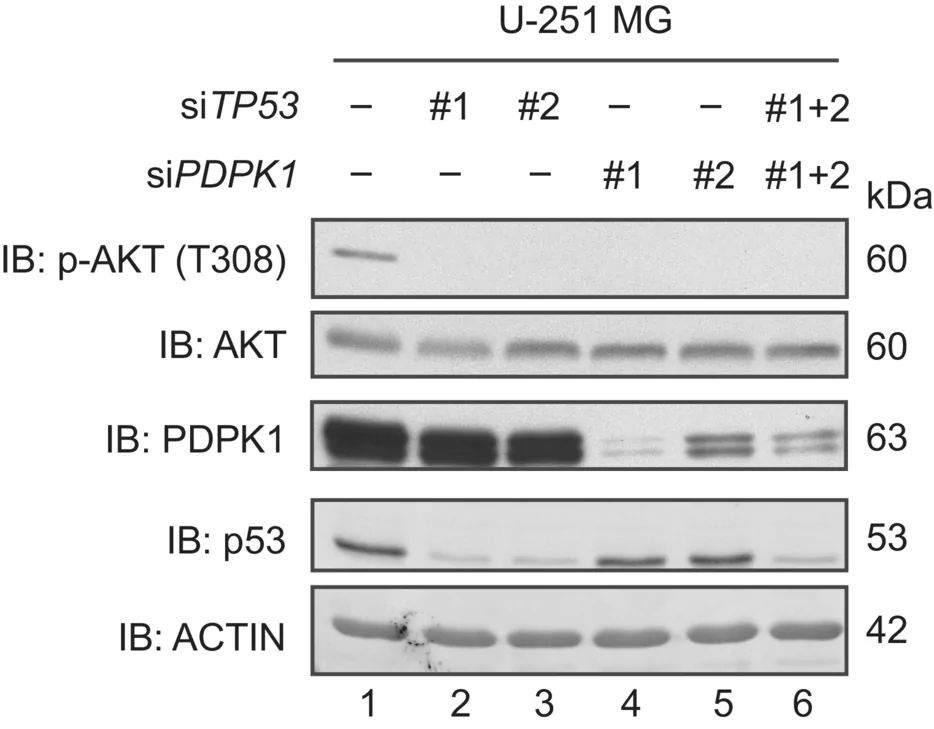
Mutant p53^R273H^ enhances PDPK1 activation. U‐251MG cells were transfected with siRNAs targeting *TP53* or *PDPK1*, either individually or in combination. Cells were lysed 48 h post‐transfection, and lysates were analyzed by western blot using the indicated antibodies (representative of *n* = 3 independent experiments).

### 3.5 PRIMA‐1^Met^ treatment disrupts p53^R273H^ ‐PDPK1 interaction

PRIMA‐1^Met^ (APR‐246) is a small molecule that can restore the DNA‐binding capacity of mutant p53 proteins and has been shown to restore wild‐type p53 functions in multiple types of cancers [46, 47, 48]. Since we showed that p53^R273H^, but not wild‐type p53, interacted with PDPK1 (Fig. 2A), we hypothesized that PRIMA‐1^Met^ treatment may induce p53 protein conformation changes, primarily through the generation of active methylene quinuclidinone (MQ) that targets Cys277 in the p53 core domain [49, 50], thereby disrupting its interaction with PDPK1. Indeed, PRIMA‐1^Met^ treatment reduced the amount of endogenous PDPK1 co‐immunoprecipitated with endogenous p53^R273H^ in a dose‐dependent manner (Fig. 5A). Furthermore, the same treatment also disrupted the close proximity between p53^R273H^ and PDPK1, as indicated by decreased PLA signals in U‐251MG cells (Fig. 5B). These data suggest that PRIMA‐1^Met^ treatment may reduce PDPK1 kinase activity by increasing PDPK1 homodimerization through the disruption of the p53^R273H^‐PDPK1 interaction. To investigate this, we first determined that 0.25 mm PRIMA‐1^Met^ was required to disrupt p53^R273H^‐PDPK1 interaction in NCI‐H1299 cells (Fig. S7). Subsequent FRET‐FLIM analysis revealed that 0.25 mm, but not 0.125 mm, of PRIMA‐1^Met^ significantly increased the PDPK1 homodimerization fraction (Fig. 5C), indicating a shift toward the less active homodimeric state.

**Fig. 5:**
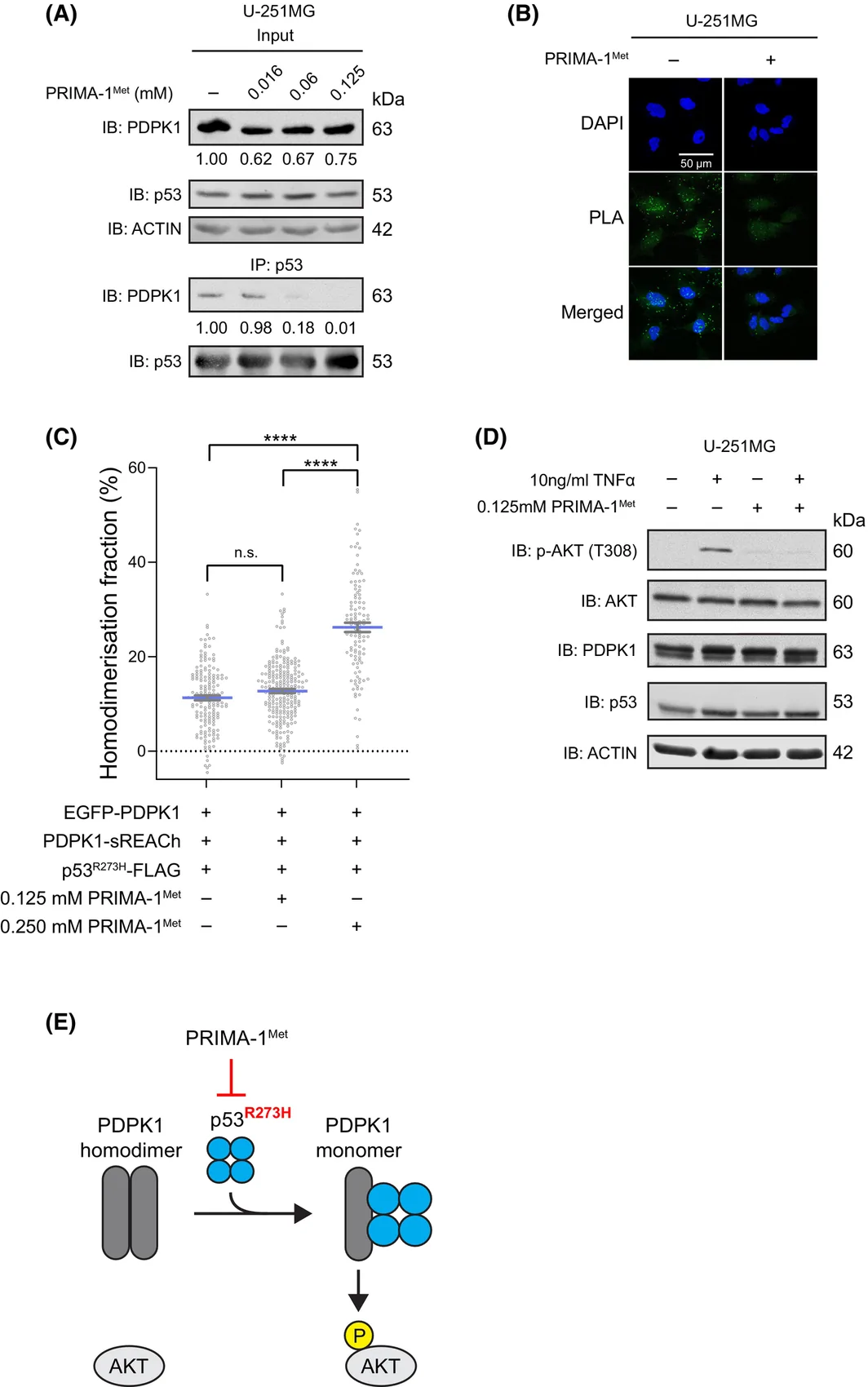
PRIMA‐1^Met^ disrupts p53^R273H^‐PDPK1 interaction. (A) U‐251MG cells were treated with the indicated doses of PRIMA‐1^Met^ for 24 h. Lysates were immunoprecipitated with anti‐p53 antibody and analyzed by western blot using the indicated antibodies (representative of *n* = 2 independent experiments). Additional validation is provided in Fig. S7. (B) Proximity ligation assay (PLA) in U‐251MG cells. Cells transfected with FLAG‐PDPK1 were fixed, permeabilized, and stained using anti‐p53 and anti‐FLAG antibodies for PLA. Confocal microscopy images show nuclei (DAPI, blue) and green fluorescence signals indicating positive PLA interactions (representative of *n* = 3 independent experiments). Scale bar = 50 μm; applies to all the images in the panel. (C) FRET‐FLIM analysis of NCI‐H1299 cells transfected with the indicated plasmids. Gray circles represent individual cells (number of cells = 171, 245, 115 from left to right). Blue horizontal lines and gray error bars represent the mean homodimerization fraction ± SEM for each group (representative of *n* = 3 independent experiments). Statistical analysis was performed using a one‐way ANOVA with *post hoc* Tukey's test. ****P value < 0.0001. (D) U‐251MG cells were treated for 24 h in DMEM containing 0.1% FBS with DMSO, TNFα, PRIMA‐1^Met^, or a combination of TNFα and PRIMA‐1^Met^. Lysates were analyzed by western blot using the indicated antibodies (representative of *n* = 2 independent experiments). (E) A schematic model illustrating the p53^R273H^‐PDPK1 interaction contributing to increased AKT phosphorylation. The p53^R273H^ mutant disrupts the inactive PDPK1 homodimer, resulting in the formation of active PDPK1 monomers that promote AKT phosphorylation. Treatment with PRIMA‐1^Met^, which has been reported to restore p53^R273H^ to a wild‐type‐like conformation, disrupts the p53^R273H^‐PDPK1 interaction and inhibits this gain‐of‐function effect of p53^R273H^.

As our data indicated that PDPK1 interacted with p53^R273H^, but not other hotspot mutants (Fig. 2C), we next sought to address the effect of PRIMA‐1^Met^ on AKT phosphorylation in the presence of different p53 hotspot mutants. To test this, we first knocked down endogenous p53^R273H^ in U‐251MG cells and subsequently performed ectopic rescue by expressing p53^R273H^, p53^R175H^, or p53^R248W^. Based on the assumption that the p53^R273H^‐PDPK1 interaction is the key driver of increased AKT phosphorylation, we initially expected that only p53^R273H^ expression would restore AKT phosphorylation. However, we observed that all three p53 mutants were able to restore AKT phosphorylation in this cell line (Fig. S8A). This result is consistent with the previous report showing the cell‐type‐specific behavior of mutant p53 [51]. To further explore this, we repeated these experiments in HCT116 p53 knockout cells. In this cell type, we found that only p53^R273H^ induced AKT phosphorylation and that PRIMA‐1^Met^ treatment dampened the p53^R273H^‐mediated AKT phosphorylation (Fig. S8B). Consistent with these findings, PRIMA‐1^Met^ treatment in U‐251MG cells reduced AKT phosphorylation levels even in the presence of TNFα, which is known to activate AKT through PDPK1 [52] (Fig. 5D, lanes 2 and 4).

Taken together, these data suggest that the p53^R273H^‐PDPK1 interaction plays a critical role in driving the activation of the AKT pathway for tumor progression and that the disruption of this interaction using PRIMA‐1^Met^ may offer a therapeutic strategy to attenuate these pathways in p53^R273H^‐expressing cancers with elevated TNFα levels (Fig. 5E).

## 4 Discussion

Several studies have shown that mutant p53 enhances activation of the PI3K/AKT pathway in cancer cells [13, 14, 15, 16], but the underlying mechanism remains unclear. We demonstrated that p53^R273H^ binds to PDPK1, an upstream kinase of AKT, and disrupts its homodimerization. This homodimerization disruption could contribute to the increased AKT phosphorylation by p53^R273H^. Therefore, we propose that the direct interaction between p53^R273H^ and PDPK1, leading to enhanced PI3K/AKT pathway activation, represents a novel and potentially impactful model.

Mutant p53 is known to be a stable and long‐lived protein compared to wild‐type p53 [5, 7]. This stability enables mutant p53 to interact with various proteins, contributing to diverse gain‐of‐function phenotypes that drive tumor progression. However, recent studies have highlighted that not all p53 mutants are the same and that they exhibit different gain‐of‐function properties [51, 53]. Our study revealed that PDPK1 specifically interacts with p53^R273H^, but not p53^WT^, p53^R175H^, or p53^R248W^, highlighting a p53^R273H^‐specific gain‐of‐function. Indeed, other studies have also reported distinct protein partners specific to each mutant p53 [54, 55]. For example, while some interacting proteins are shared between p53^R273H^ and p53^WT^, Coffill *et al*. showed that NRD1 specifically interacts with p53^R273H^ [54]. Xiong *et al*. similarly identified p53^R273H^‐specific interacting proteins that did not bind to p53^R175H^ or p53^R248W^ [55]. In addition to these unique interactomes for each mutant p53, cell‐type specificity also significantly influences mutant p53 functions [51]. This functional complexity has been documented. For instance, ectopic expression of several p53 mutants (R175H, R248W, and R273H) has been shown to increase AKT phosphorylation in certain contexts [16], while other studies found that only p53^R273H^ depletion (and not depletion of other mutants) reduced AKT phosphorylation [15]. These recent studies, including ours, suggest that individual mutant p53 proteins may employ distinct mechanisms to activate AKT signaling, highlighting the mutant‐specific and context‐dependent nature of gain‐of‐function activities. Despite these complexities, our study highlights a unique mechanism of AKT activation mediated by p53^R273H^ through PDPK1.

Our *in silico* modeling revealed a potential explanation for this specific interaction. Our MD simulation indicated that the R273H mutation exposes a hydrophobic patch (109FRLGFL114) within the p53 DBD, which could potentially facilitate binding to PDPK1. Furthermore, our MD simulation showed that the introduction of the F270A mutation in p53^R273H^ (generating the double mutant F270A/R273H) restores this hydrophobic patch to a buried position within the DBD core, similar to that of wild‐type p53. Despite having the intact hydrophobic patch, the p53^F270A/R273H^ failed to bind PDPK1, highlighting the importance of the exposed hydrophobic patch in p53^R273H^. These observations suggest that the increased hydrophobic surface of p53^R273H^, specifically the six‐amino‐acid hydrophobic patch (FRLGFL), could be a key for p53^R273H^ to bind to PDPK1. In addition to increased hydrophobicity, p53^R273H^ has also been reported to exhibit a higher propensity for aggregation compared to wild‐type p53 [56]. However, we demonstrated that p53^R175H^, a well‐characterized structural mutant known to form aggregates [57, 58], does not interact with PDPK1. Therefore, the exposed hydrophobic patch, rather than protein aggregation, is likely the significant factor in p53^R273H^ binding to PDPK1. To further understand the nature of p53^R273H^ binding, we performed *in silico* docking using the program HADDOCK (High Ambiguity Driven biomolecular Docking) to model the interaction between PDPK1 and p53^R273H^ DBD. The model revealed both hydrophobic and electrostatic interactions between p53^R273H^ DBD and PDPK1. Notably, the side chains of F113 and L114 at the hydrophobic patch of p53^R273H^ DBD engaged with the hydrophobic surface of PDPK1 (Fig. S9). These data further support the notion that the hydrophobic patch is important for the direct interaction between p53^R273H^ and PDPK1. Nevertheless, the directly interacting domains remain to be elucidated by X‐ray crystallography or cryo‐electron microscopy (cryo‐EM).

Recent studies have shown that PDPK1 homodimer dissociation is required for PDPK1 to interact with and phosphorylate its substrate, such as AKT [41, 42]. We demonstrated, for the first time, that p53^R273H^ interacts with PDPK1 and reduces the PDPK1 homodimerization fraction, suggesting that the physical interaction between p53^R273H^ and PDPK1 promotes the dissociation of the PDPK1 homodimer. Consistent with this, we showed that the PDPK1‐binding deficient p53^F270A/R273H^ did not dissociate the PDPK1 homodimer. Consequently, cancer cells expressing p53^R273H^ likely maintain a higher proportion of monomeric PDPK1, which is the active form capable of phosphorylating AKT. Supporting this notion, we showed that the depletion of p53^R273H^ in U‐251MG cells led to decreased AKT T308 phosphorylation, despite the presence of PDPK1, the primary upstream kinase for this residue [44, 45]. Furthermore, PDPK1 knockdown reduced AKT T308 phosphorylation even in the presence of p53^R273H^, indicating that any PDPK1‐independent contribution of p53^R273H^ to T308 phosphorylation is minimal, if not negligible. We also demonstrated, by using an *in vitro* kinase assay, that recombinant p53^R273H^ yielded only a marginal increase in PDPK1 kinase activity. This modest effect likely stems from the absence of essential cellular factors, such as membrane localization. Previous studies have shown that the cotranslocation of PDPK1 and AKT to the plasma membrane is critical for efficient AKT phosphorylation [41, 59]. This suggests that the spatial and biochemical complexities of the cellular environment are necessary to fully recapitulate the p53^R273H^‐dependent AKT activation observed in cells. Taken together, these findings support a model in which p53^R273H^‐PDPK1 interaction shifts the equilibrium toward active PDPK1 monomers to drive AKT signaling.

Elucidating whether p53^R273H^ directly influences the PDPK1‐AKT interaction remains an important question. However, as previously reported by Ding *et al*. [60], detecting the endogenous interaction between PDPK1 and AKT is difficult without specialized techniques, such as protein fragment complementation (IFPN/IFPC), likely due to the highly transient nature of this kinase‐substrate interaction. While our current findings demonstrate that p53^R273H^ modulates AKT signaling via its interaction with PDPK1, further studies utilizing advanced approaches to assess the effect of p53^R273H^ on the real‐time dynamics of the PDPK1‐AKT complex are warranted.

In the tumor microenvironment, inflammatory cytokines such as TNFα play an important role in regulating immune infiltration, tumor growth, and response to therapy [61]. TNFα, a major regulator of inflammation, is known to activate the PI3K/AKT pathway, which subsequently stimulates the NF‐κB pathway to upregulate target genes [62, 63]. Notably, p53^R273H^ has been reported to increase cancer cell responsiveness to TNFα and enhance their invasive potential by further stimulating the NF‐κB pathway [38]. Interestingly, p53^R273H^ has also been shown to activate the PI3K/AKT pathway, inducing metastasis in a breast cancer model [14, 15]. Furthermore, p53^R273H^ is thought to stimulate the PI3K/AKT pathway through receptor recycling [14]. These studies suggest a potential link between the regulation of the PI3K/AKT pathway by p53^R273H^ and the cellular responses to TNFα stimulation. Consistent with this, we demonstrated that PRIMA‐1^Met^ treatment, which disrupted the p53^R273H^‐PDPK1 interaction, reduced the TNFα‐mediated AKT phosphorylation. Our data highlight the potential of PRIMA‐1^Met^ as a novel therapeutic option for p53^R273H^‐expressing cancers with elevated TNFα levels.

## 5 Conclusion

Our study reveals a novel mechanism by which mutant p53^R273H^ promotes AKT phosphorylation through a direct interaction with PDPK1. Mechanistically, this interaction disrupts inhibitory PDPK1 homodimerization and promotes PDPK1‐mediated AKT phosphorylation. Finally, PRIMA‐1^Met^ treatment disrupts the p53^R273H^‐PDPK1 interaction and suppresses downstream AKT phosphorylation, highlighting this interaction as a potential new therapeutic target in cancers harboring the *TP53* ^*R273H*^ mutation.

## Conflict of interest

The authors declare no conflict of interest.

## Author contributions

MCL, AKG, YI, MO, TYWT, WY, and SRK performed the experiments. MCL, AKG, YI, and KI designed the experiments and analyzed the results. JN and VG performed and analyzed the FRET/FLIM experiments. SK and CSV performed and analyzed the *in silico* MD simulations and docking experiments. MCL, AKG, SK, JN, CSV, YI, and KI wrote the manuscript. All authors reviewed and approved the final manuscript and agree to be accountable for all aspects of the work.

## Supporting information


**Fig. S1.** p53^R273H^ interacts with PDPK1.

**Fig. S2.** Western blot and immunofluorescence detection of endogenous PDPK1 in U‐251MG cells.

**Fig. S3.** Immunofluorescence staining of p53^R273H^‐FLAG and p53^F270A/R273H^‐FLAG in SaOS‐2 cells.

**Fig. S4.** Generation of U‐251MG p53 knockout (p53KO) cells.

**Fig. S5.** Proximity Ligation Assay (PLA) of FLAG‐PDPK1 and different mutant p53 in U‐251MG p53 knockout cells.

**Fig. S6.** *In vitro* PDPK1 kinase assay.

**Fig. S7.** p53^R273H^‐PDPK1 interaction was disrupted using 0.25 mM of PRIMA‐1^Met^ in NCI‐H1299 cells.

**Fig. S8.** Ectopic expression of different p53 mutants in cancer cell lines.

**Fig. S9.** A model showing the *in silico* docking simulation of the predicted p53^R273H^ DBD‐PDPK1 complex.

**Table S1.** CRAPome analysis of the twelve p53^R273H^ interacting proteins in the PI3K‐AKT signaling pathway enriched by KEGG pathway analysis.

## Data Availability

The data and materials reported in this article are available from the corresponding author upon reasonable request.
