# Supplementary material for: Mutant p53R273H disrupts PDPK1 homodimerization and contributes to PDPK1 activation
Source: Mol Oncol. 2026 Sep 29:10.1002/1878-0261.70341. Online ahead of print. doi: 10.1002/1878-0261.70341 (PMC13621434; doi:10.1002/1878-0261.70341)
Supplement: Supplementary file 1 [file MOL2-9999-0-s001.pdf]

Supplementary Figure 3

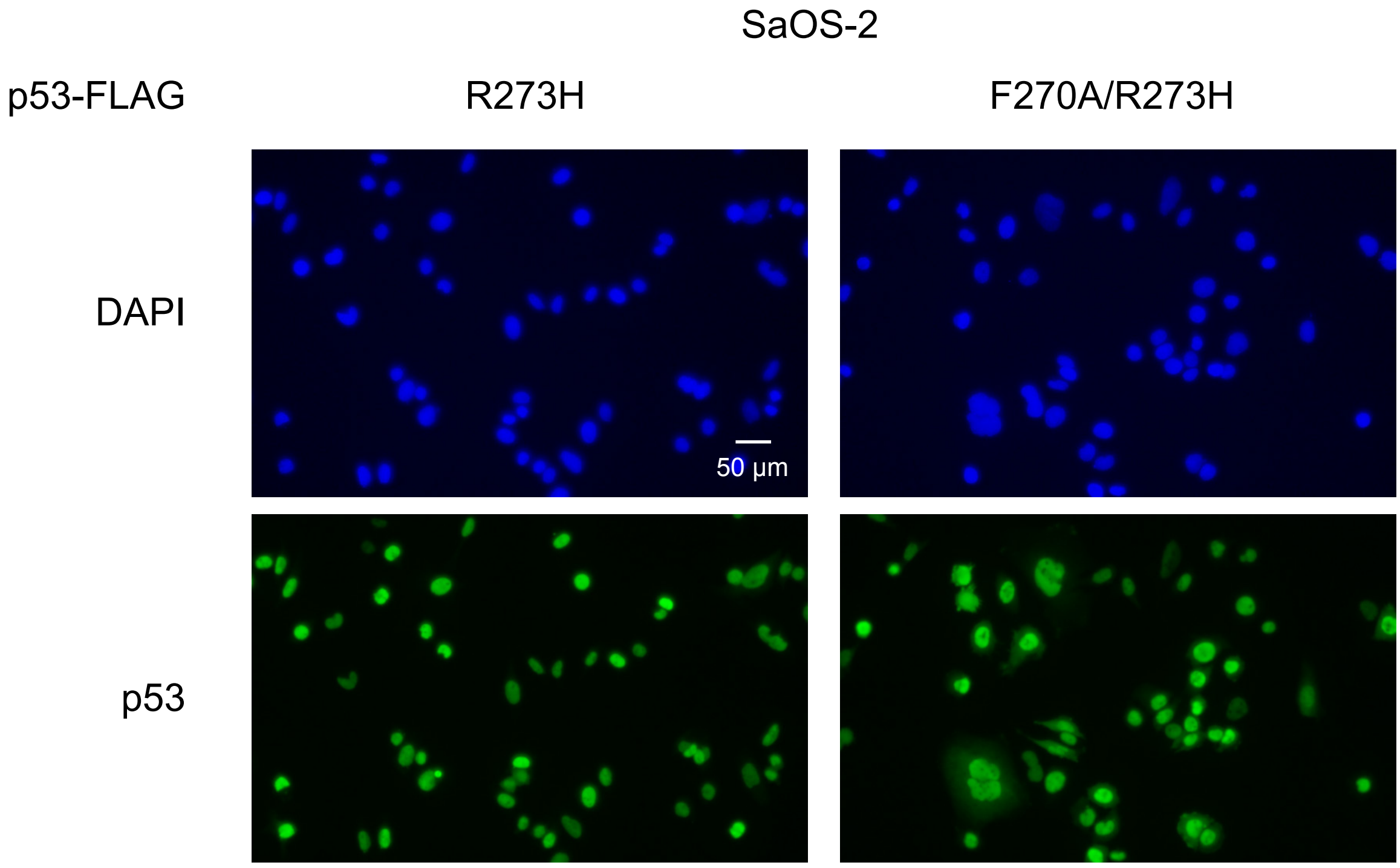

Supplementary Figure 3. Immunofluorescence staining of p53^{R273H}-FLAG and p53$^{F270A/R273H}$-FLAG in SaOS-2 cells. SaOS-2 cells transfected with p53^{R273H}-FLAG or p53$^{F270A/R273H}$-FLAG were fixed, permeabilized, and stained using anti-p53 antibody (n = 1 experiment). Fluorescence micrographs showing localization of both p53^{R273H} and p53$^{F270A/R273H}$ in the nucleus. The nucleus is stained by DAPI (in blue). Scale bar = 50 µm (applies to all the images in the panel).
