# Supplementary material for: Mutant p53R273H disrupts PDPK1 homodimerization and contributes to PDPK1 activation
Source: Mol Oncol. 2026 Sep 29:10.1002/1878-0261.70341. Online ahead of print. doi: 10.1002/1878-0261.70341 (PMC13621434; doi:10.1002/1878-0261.70341)
Supplement: Supplementary file 2 [file MOL2-9999-0-s002.pdf]

# Supplementary Figure 8

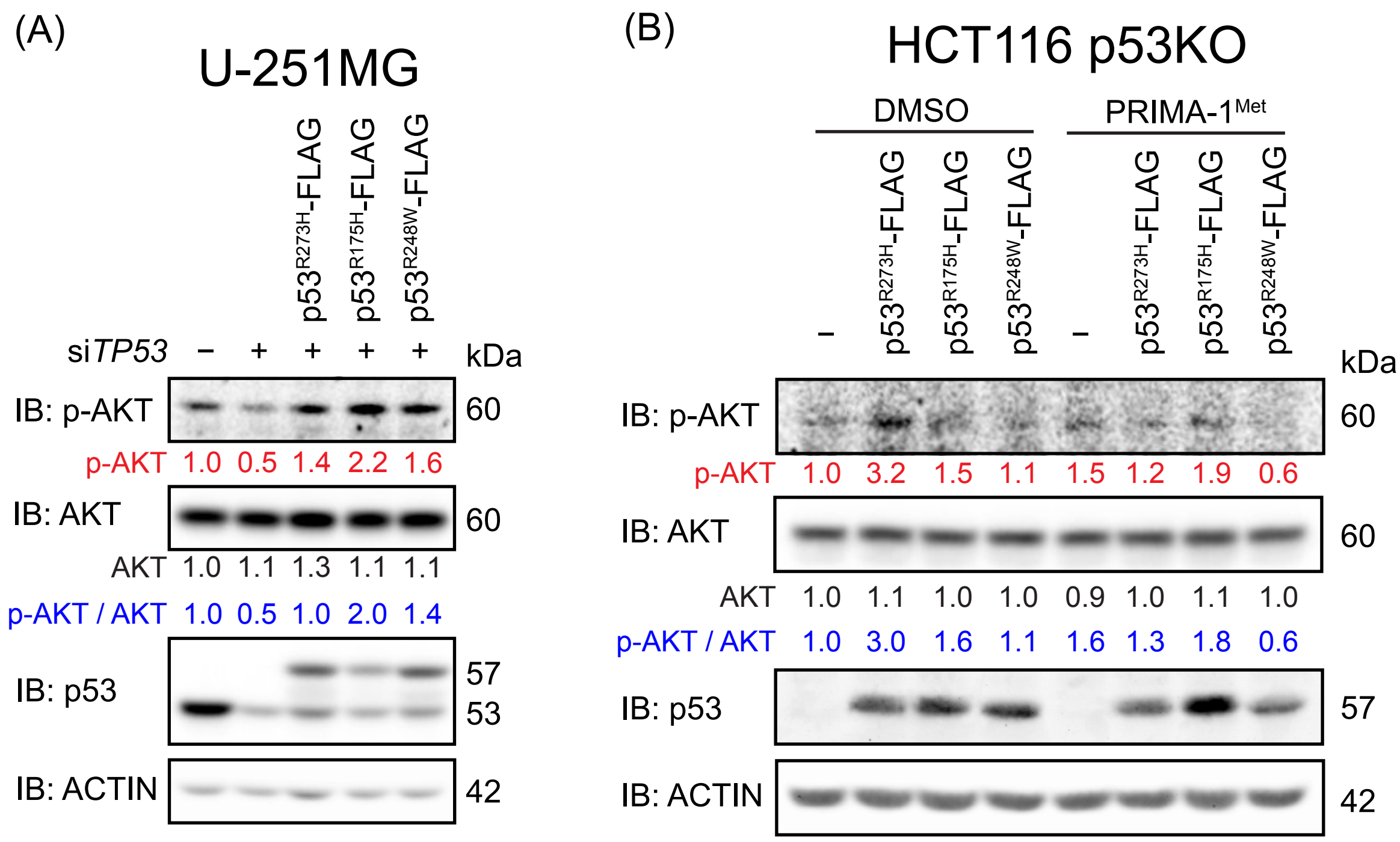


**Supplementary Figure 8. Ectopic expression of different p53 mutants in cancer cell lines.** (A) U-251MG cells were transfected with control or *TP53*-targeting siRNAs together with empty vector or plasmids expressing the indicated p53 mutants. Cell lysates were subjected to Western blot using the indicated antibodies (representative of n = 2 independent experiments). (B) HCT116 p53 knockout (KO) cells were transfected with empty vector or the indicated p53 mutant expressing plasmids and treated with either DMSO or PRIMA-$1^{Met}$ (0.125 mM) for 24 hours. Cell lysates were subjected to Western blot using the indicated antibodies (representative of n = 2 independent experiments). Densitometric quantification was performed using Image J (p-AKT in red, total AKT in black, p-AKT / total AKT ratio in blue).
