# Supplementary material for: Mutant p53R273H disrupts PDPK1 homodimerization and contributes to PDPK1 activation
Source: Mol Oncol. 2026 Sep 29:10.1002/1878-0261.70341. Online ahead of print. doi: 10.1002/1878-0261.70341 (PMC13621434; doi:10.1002/1878-0261.70341)
Supplement: Supplementary file 3 [file MOL2-9999-0-s003.pdf]

Supplementary Figure 4

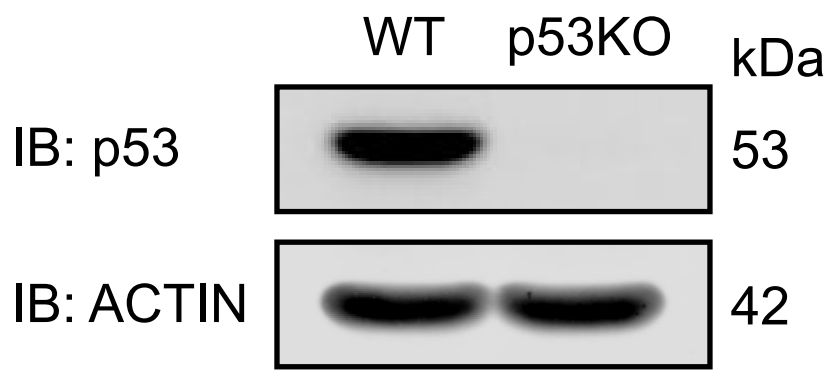

Supplementary Figure 4. Generation of U-251MG p53 knockout (p53KO) cells. p53KO U-251MG cells were generated using Cas9 Ribonucleoprotein (RNP) complex. Cell lysates were subjected to Western blot analysis using the indicated antibodies to verify the knockout (representative of n = 3 independent experiments).
