# Supplementary material for: Mutant p53R273H disrupts PDPK1 homodimerization and contributes to PDPK1 activation
Source: Mol Oncol. 2026 Sep 29:10.1002/1878-0261.70341. Online ahead of print. doi: 10.1002/1878-0261.70341 (PMC13621434; doi:10.1002/1878-0261.70341)
Supplement: Supplementary file 4 [file MOL2-9999-0-s004.pdf]

Supplementary Figure 5

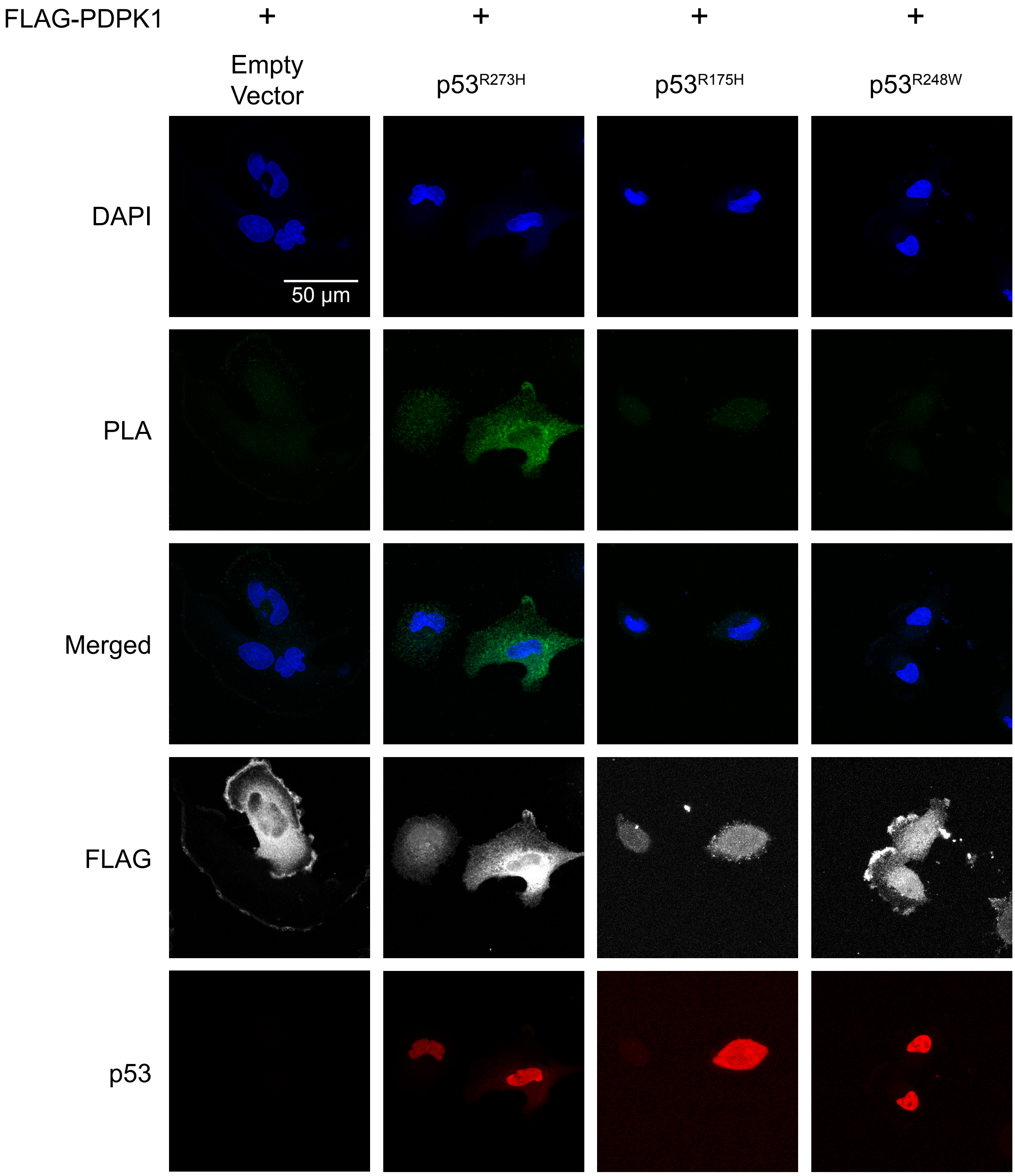

Supplementary Figure 5. Proximity Ligation Assay (PLA) of FLAG-PDPK1 and different mutant p53 in U-251MG p53 knockout cells. Cells co-transfected with FLAG-PDPK1 and different mutant p53 ($p53^{R273H}$, $p53^{R175H}$ or $p53^{R248W}$) were fixed, permeabilized, and stained using anti-p53 and anti-FLAG antibodies for PLA (representative of n = 2 independent experiments). Confocal microscopy images show FLAG-PDPK1 (grey), mutant p53 (red), nuclei (DAPI, blue), and green fluorescence signals indicating positive PLA interactions. Scale bar = 50 µm (applies to all the images in the panel).
