# Supplementary material for: Mutant p53R273H disrupts PDPK1 homodimerization and contributes to PDPK1 activation
Source: Mol Oncol. 2026 Sep 29:10.1002/1878-0261.70341. Online ahead of print. doi: 10.1002/1878-0261.70341 (PMC13621434; doi:10.1002/1878-0261.70341)
Supplement: Supplementary file 5 [file MOL2-9999-0-s006.pdf]

Supplementary Figure 6

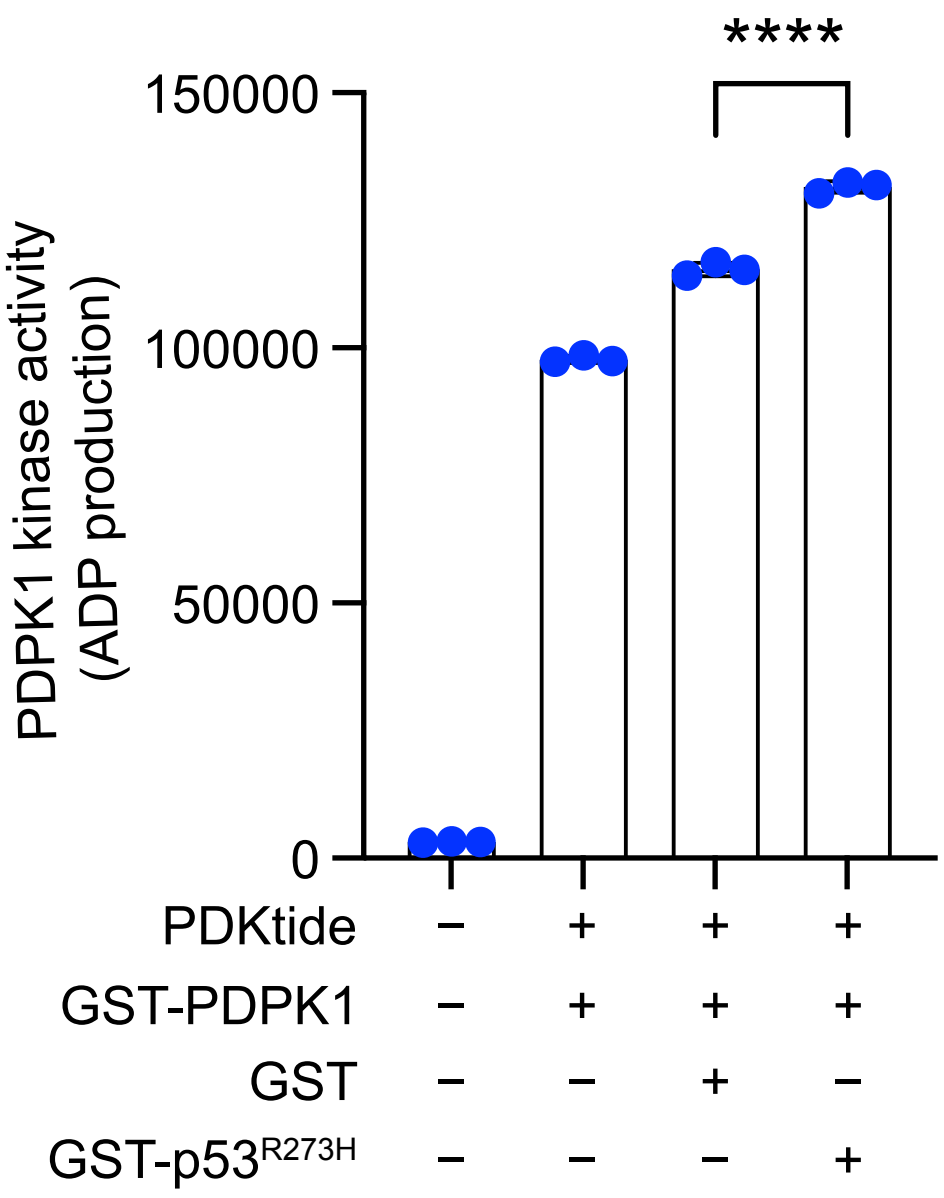

Supplementary Figure 6. *In vitro* PDPK1 kinase assay. Recombinant GST-PDPK1 was pre-incubated with GST or GST-p53^{R273H} on ice for 1 hour before the addition of PDPK1 substrate (PDKtide) and ATP to start the kinase reaction. ADP production was quantified to determine PDPK1 kinase activity (n = 2 independent experiments). Bars represent mean PDPK1 kinase activity ± SD of technical triplicates from a representative experiment. ****P value < 0.0001. Statistical analysis was done by two-tailed unpaired Student's t-test.
