# Supplementary material for: Mutant p53R273H disrupts PDPK1 homodimerization and contributes to PDPK1 activation
Source: Mol Oncol. 2026 Sep 29:10.1002/1878-0261.70341. Online ahead of print. doi: 10.1002/1878-0261.70341 (PMC13621434; doi:10.1002/1878-0261.70341)
Supplement: Supplementary file 6 [file MOL2-9999-0-s007.pdf]

Supplementary Figure 2

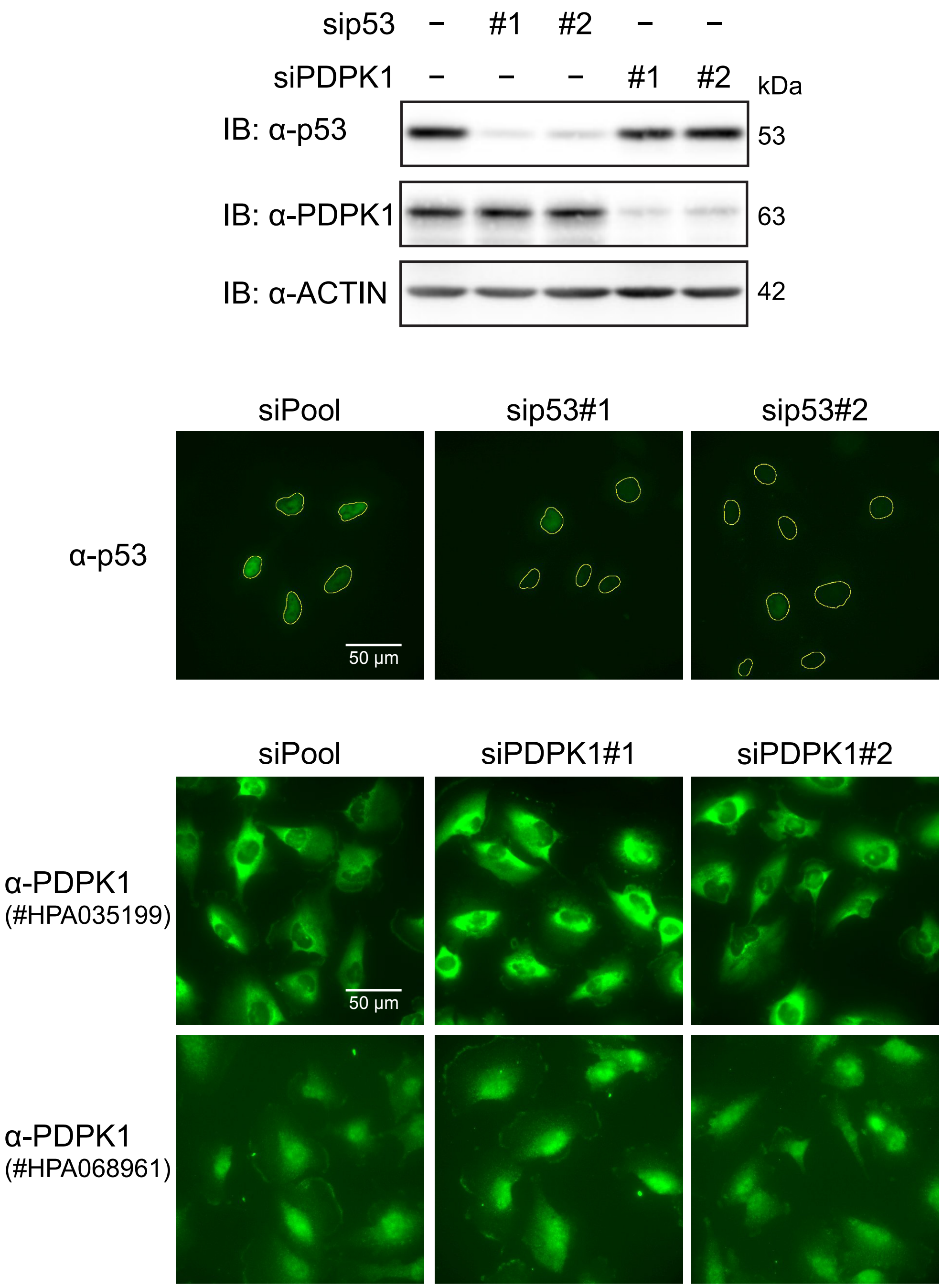

Supplementary Figure 2. Western blot and immunofluorescence detection of endogenous PDPK1 in U-251MG cells. Lysates of U-251MG cells transfected with the indicated siRNAs targeting *TP53* or *PDPK1* were subjected to Western blot, confirming successful knockdown of the respective proteins (representative of n = 3 independent experiments). Cells were also fixed, permeabilized, and stained using anti-p53 or anti-PDPK1 antibodies (representative of n = 2 independent experiments). Fluorescence micrographs revealed reduced p53 fluorescence signals in p53 knockdown cells but no significant change in PDPK1 signal in PDPK1 knockdown cells. This suggests that the PDPK1 antibodies are unable to detect endogenous PDPK1 levels by immunofluorescent staining. Yellow outlines indicate the nuclei of cells stained with anti-p53 antibody. Scale bar = 50 µm (applies to all the images in the panel).
