# Supplementary material for: Mutant p53R273H disrupts PDPK1 homodimerization and contributes to PDPK1 activation
Source: Mol Oncol. 2026 Sep 29:10.1002/1878-0261.70341. Online ahead of print. doi: 10.1002/1878-0261.70341 (PMC13621434; doi:10.1002/1878-0261.70341)
Supplement: Supplementary file 7 [file MOL2-9999-0-s008.pdf]

Supplementary Figure 1

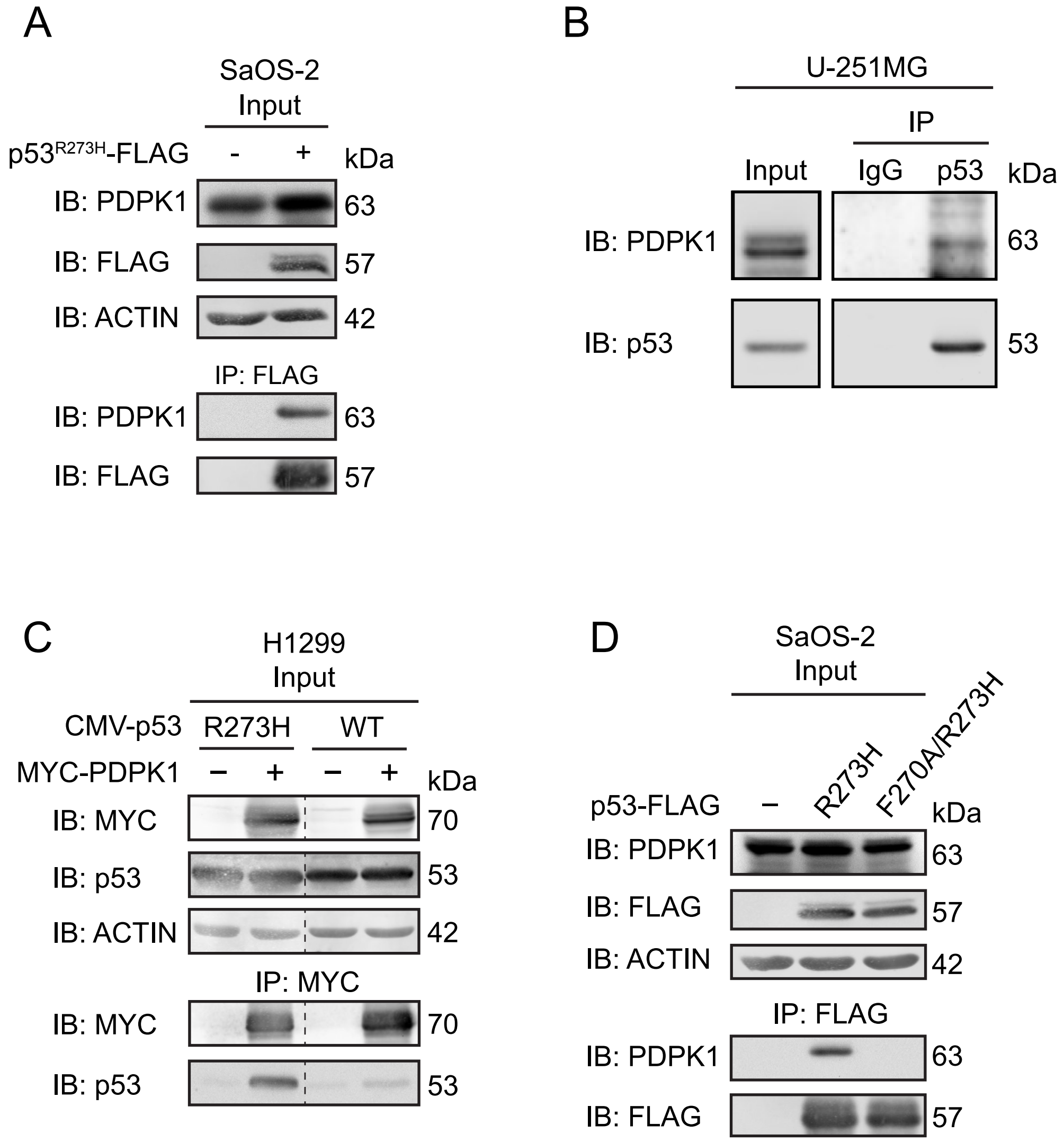

Supplementary Figure 1. p53^{R273H} interacts with PDPK1. (A) Lysates of SaOS-2 cells transfected with an empty vector expressing the FLAG tag alone or p53^{R273H}-FLAG were subjected to co-immunoprecipitation using anti-p53 antibody, followed by Western blot with the indicated antibodies (representative of n = 2 independent experiments). (B) Lysate of U-251MG cells was subjected to co-immunoprecipitation using goat IgG control antibody or anti-p53 antibody, followed by Western blot with the indicated antibodies (n = 1 experiment). (C) Lysates of H1299 cells transfected with an empty vector expressing the MYC tag alone or MYC-PDPK1, together with the indicated p53 constructs, were subjected to co-immunoprecipitation using anti-MYC antibody, followed by Western blot with the indicated antibodies (n = 1 experiment). Vertical dashed lines indicate non-adjacent lanes from the same blot grouped together under identical exposure conditions. (D) Lysates of SaOS-2 cells transduced with adenoviruses expressing the indicated p53 constructs were immunoprecipitated using anti-FLAG antibody, followed by Western blot with the indicated antibodies (n = 1 experiment).
