# Supplementary material for: Mutant p53R273H disrupts PDPK1 homodimerization and contributes to PDPK1 activation
Source: Mol Oncol. 2026 Sep 29:10.1002/1878-0261.70341. Online ahead of print. doi: 10.1002/1878-0261.70341 (PMC13621434; doi:10.1002/1878-0261.70341)
Supplement: Supplementary file 8 [file MOL2-9999-0-s009.pdf]

Supplementary Figure 7

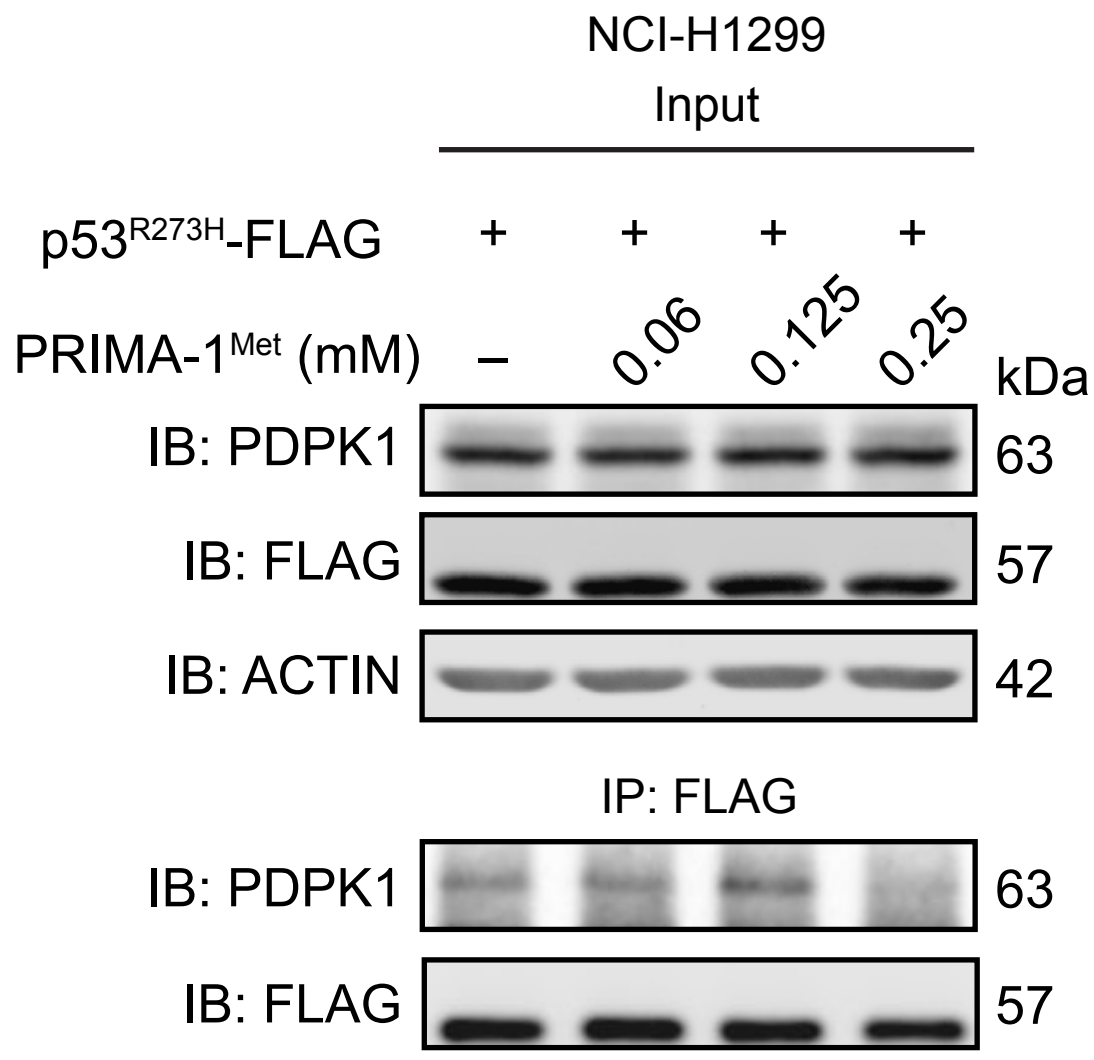

Supplementary Figure 7. p53^{R273H}-PDPK1 interaction was disrupted using 0.25 mM of PRIMA-1^{Met} in NCI-H1299 cells. NCI-H1299 cells transfected with p53^{R273H}-FLAG were treated with the indicated dose of PRIMA-1^{Met} for 24 hours. Lysates were immunoprecipitated with anti-FLAG antibody and analyzed by Western blot using the indicated antibodies (n = 1 experiment).
