# Supplementary material for: Mutant p53R273H disrupts PDPK1 homodimerization and contributes to PDPK1 activation
Source: Mol Oncol. 2026 Sep 29:10.1002/1878-0261.70341. Online ahead of print. doi: 10.1002/1878-0261.70341 (PMC13621434; doi:10.1002/1878-0261.70341)
Supplement: Supplementary file 9 [file MOL2-9999-0-s010.pdf]

Supplementary Figure 9

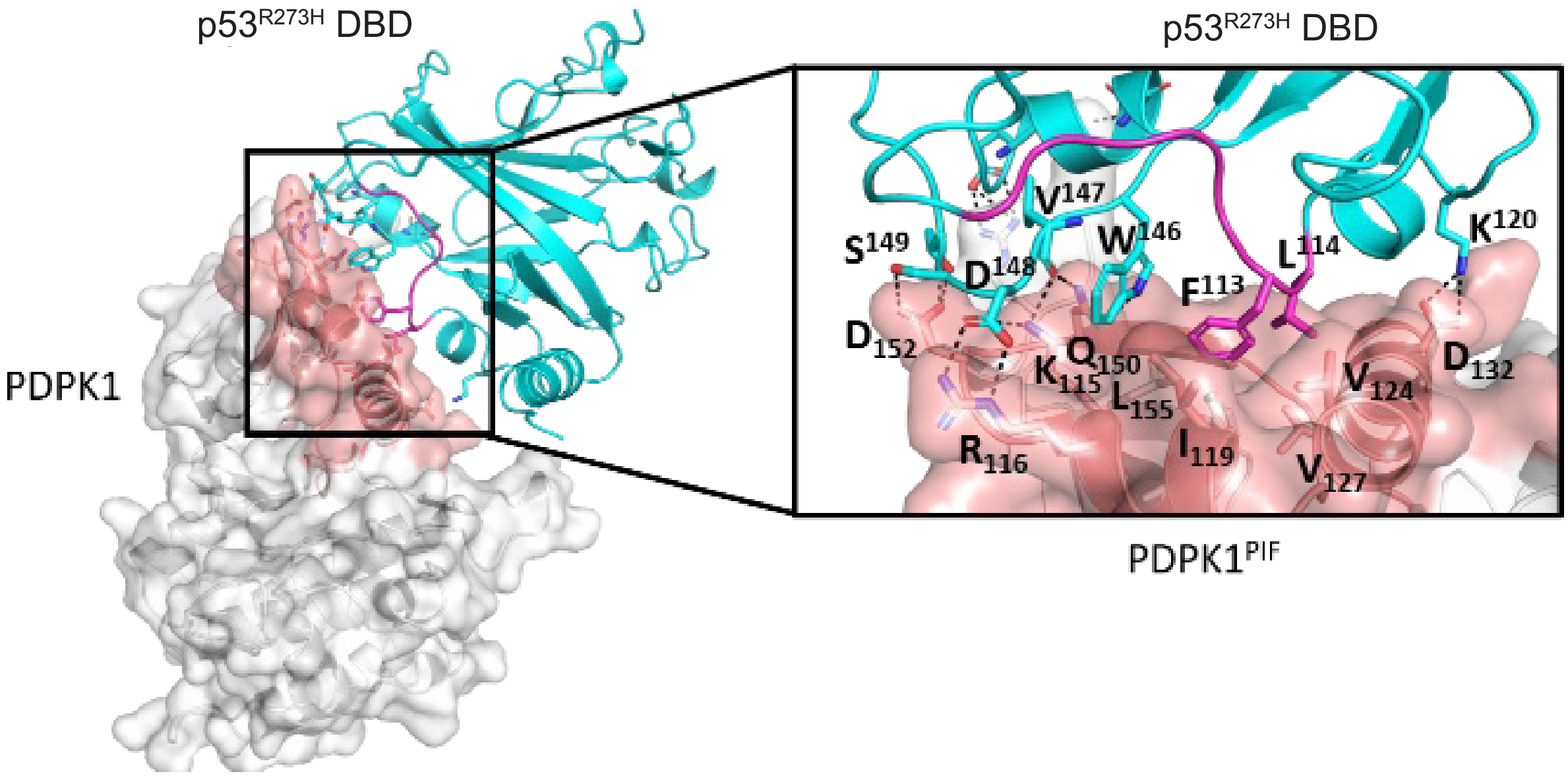

Supplementary Figure 9. A model showing the *in silico* docking simulation of the predicted p53^{R273H} DBD-PDPK1 complex. p53^{R273H} DBD is shown in cyan, with the hydrophobic patch (in magenta), exposed to solvent during the simulation. Interacting residues are shown as sticks (PDPK1 and p53^{R273H} DBD residues labelled in subscript and superscript, respectively), and interactions are indicated by black dotted lines. PDPK1 PIF domain (PDPK1^{PIF}) is shown in pink.
