# Supplementary material for: Mutant p53R273H disrupts PDPK1 homodimerization and contributes to PDPK1 activation
Source: Mol Oncol. 2026 Sep 29:10.1002/1878-0261.70341. Online ahead of print. doi: 10.1002/1878-0261.70341 (PMC13621434; doi:10.1002/1878-0261.70341)
Supplement: Supplementary file 10 [file MOL2-9999-0-s005.docx]

**Supplementary Table 1. CRAPome analysis of the twelve p53^R273H^ interacting proteins in the PI3K-AKT signaling pathway enriched by KEGG pathway analysis.** The twelve TP53^R273H^ interacting proteins were subjected to CRAPome analysis (<https://reprint-apms.org/?q=chooseworkflow>) to obtain the CRAPome Score. The CRAPome Score represents the number of experiments, out of a total of 411, in which the indicated protein was detected. Lower CRAPome score indicates specific interacting protein or protein with low abundance. Brief description of the proteins obtained from UniProtKB/Swiss-Prot Summary for each genes using GeneCards^®^: The Human Gene Database (https://www.genecards.org/).

|  | Protein | CRAPome  Score | Description |
| --- | --- | --- | --- |
| 1 | PDPK1 | 0/411 | Serine/threonine kinase, master kinase for subgroup of AGC family protein kinases including AKT |
| 2 | MDM2 | 0/411 | E3 ubiquitin-protein ligase, ubiquitination of p53 leading to its degradation by proteosome |
| 3 | GRB2 | 7/411 | Non-enzymatic adapter protein, dock onto activated cell surface receptors and interact with SOS1 signal transduction |
| 4 | THBS1 | 8/411 | Adhesive glycoprotein, mediates cell-to-cell and cell-to-matrix interactions |
| 5 | MAPK1 | 15/411 | Serine/threonine kinase, essential component of the MAPK/ERK signal transduction pathway |
| 6 | TP53 | 52/411 | Multifunctional transcription factor, induces cell cycle arrest, DNA repair or apoptosis to suppress tumors |
| 7 | GNB2 | 63/411 | Guanine nucleotide-binding proteins (G proteins), modulator or transducer in various transmembrane signaling systems |
| 8 | PPP2CA | 84/411 | Catalytic subunit of protein phosphatase 2A (PP2A), functions as serine/threonine phosphatase for wide variety of proteins |
| 9 | YWHAQ | 153/411 | Adapter protein, binds to phosphoserine or phosphothreonine motif of many proteins, negatively regulates PDPK1 activity |
| 10 | YWHAZ | 180/411 | Adapter protein, binds to phosphoserine or phosphothreonine motif of many proteins |
| 11 | HSP90AA1 | 272/411 | Heat Shock Protein 90 Alpha Family Class A Member 1, molecular chaperone that promotes maturation, structural maintenance and proper regulation of target proteins |
| 12 | HSP90AB1 | 277/411 | Heat Shock Protein 90 Alpha Family Class B Member 1, molecular chaperone that promotes maturation, structural maintenance and proper regulation of target proteins |
